# Interrogating the structure and function of the human voltage-gated proton channel (hH_v_1) with a fluorescence noncanonical amino acid

**DOI:** 10.1101/2025.10.23.684235

**Published:** 2025-10-24

**Authors:** Emerson M. Carmona, William N. Zagotta, Sharona E. Gordon

**Affiliations:** Department of Neurobiology and Biophysics, University of Washington, Seattle, WA, U.S.

## Abstract

The human voltage-gated proton channel (hH_v_1) is a dimer of voltage-sensor domains (VSDs) containing highly selective proton permeation pathways in each monomer. In addition to voltage, hH_v_1 is regulated by other stimuli, including pH gradients, mechanical forces, and ligands such as Zn^2+^. Aside from the VSDs, this membrane protein contains an N-terminal domain and a C-terminal coiled-coil domain (CC) formed between the monomers. To address the need for direct measurements of conformational rearrangements in hH_v_1, we developed a Förster resonance energy transfer (FRET) approach to measuring the conformational rearrangements in full-length hH_v_1 purified from *E. coli*. We used genetic code expansion (GCE) to generate a library of 14 full-length hH_v_1 constructs, each incorporating the fluorescent noncanonical amino acid acridon-2-ylalanine (Acd) at a different site throughout the various structural domains. Following the expression and purification of these hH_v_1-Acd proteins, we found that 12 sites yielded stable and functional proton-permeable channels. The fluorescence properties of Acd at each site showed small site-specific differences. Furthermore, we measured site-specific FRET efficiencies from tryptophan (Trp) and tyrosine (Tyr) to Acd in the hH_v_1-Acd proteins and found results consistent with correct folding in detergent micelles. Notably, the addition of Zn^2+^ produced reversible changes in FRET, with affected residues clustered on the intracellular side of the channel.

## Introduction

The human voltage-gated proton channel (hH_v_1) is a membrane protein that forms a highly selective proton channel (1, 2). Despite its physiological importance, its molecular mechanisms are still poorly understood, including the precise structural identity of the permeation pathway and the gating mechanism of voltage (3, 4), pH gradients (3, 5), membrane stretch (6), and ligands such as the classical inhibitor Zn^2+^ (7, 8). Understanding these processes requires connecting the functional properties to the hH_v_1 structure.

Each monomer of hH_v_1 dimers includes an intracellular N-terminal domain of unknown function, a transmembrane voltage-sensor domain (VSD), and an intracellular coiled-coil (CC), which forms the primary intersubunit interface (9, 10). Current structural models, however, have been obtained from truncated or chimeric channel constructs (11, 12), and thus, it is not clear whether they correspond to physiological or functional conformations. Moreover, the multiple kinetic components of H_v_1 gating currents suggest that the protein’s resting state comprises an ensemble of conformations (4, 5), which is consistent with the conformational flexibility inferred from structural approaches (12–14). Because protein function arises from this distribution of conformational states (15), approaches to directly measure structural heterogeneity in the full-length hH_v_1 are needed.

An attractive approach to interrogate the structural heterogeneity of hH_v_1 is fluorescence spectroscopy, which offers high sensitivity at nanomolar protein concentrations and compatibility with near-physiological conditions. Fluorescence can report on local environment and accessibility changes, as well as quantify distance changes through Förster resonance energy transfer (FRET) (16). Importantly, time-resolved FRET measured using lifetimes preserves information about conformational heterogeneity on the nanosecond timescale, which is lost in steady-state measurements due to the averaging process (17). Together, these capabilities make fluorescence, especially time-resolved FRET, a powerful method to connect hH_v_1 structure and function (18).

**Figure 1.**
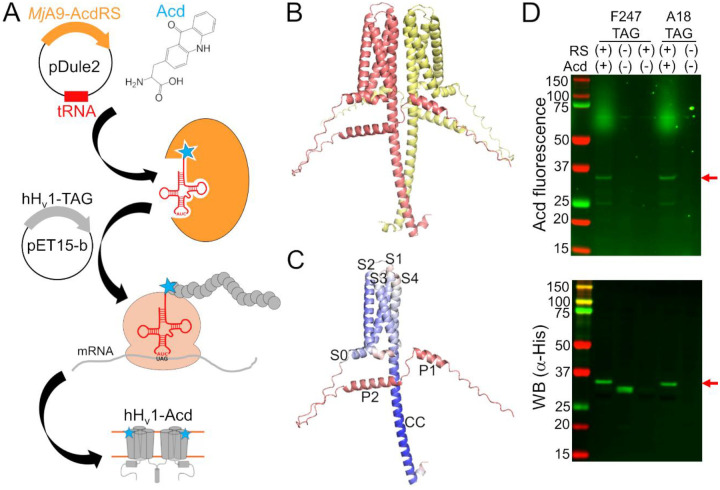
hH_v_1 was labeled with the fluorescent noncanonical amino acid Acd by genetic code expansion. (A) Cartoon showing the components of the genetic code expansion system used to incorporate Acd via amber codon suppression in hH_v_1. (B) AlphaFold structural model of the dimer hH_v_1 colored by subunit. (C) AlphaFold structural model of a single hH_v_1 subunit colored by the predicted Local Distance Difference Test (pLDDT). Model confidence was represented by a gradient from red to blue, indicating low to high pLDDT. (D) In-gel fluorescence and Western blot to evaluate Acd incorporation. An Acd fluorescence band corresponding to the molecular weight of full-length hH_v_1 (red arrow) was observed in cellular extracts after separation by SDS-PAGE only when cells contained the *Mj*A9-AcdRS/tRNA plasmid (RS) and were grown in the presence of 1mM Acd (top). The Western blot against the His-tag present at the N-terminus of hH_v_1 confirmed that the band corresponded to the recombinant protein (bottom). Note the hH_v_1 truncated product produced when the amber stop codon is replacing F247 in the absence of the aminoacyl-tRNA synthetase/tRNA plasmid and Acd.

A few obstacles remain to directly measuring conformational heterogeneity of hH_v_1. First, a method to express and purify stable and functional protein is needed, which is challenging for membrane proteins. Second, site-specific fluorophore labeling is difficult at buried sites, resulting in low labeling efficiency. Third, traditional fluorophores are bulky and have long linkers (14), resulting in protein structure perturbation and heterogeneity dominated by the properties of the label (19). A recent method was optimized for robust expression and purification of full-length, functional hH_v_1 in *E. coli* (20, 21). The remaining obstacles can be overcome by labeling the protein with the fluorescent noncanonical amino acid acridon-2-ylalanine (Acd) using genetic code expansion (GCE) (22–24). This technology allows site-specific labeling during translation by reassigning an amber stop codon to an orthogonal aminoacyl-tRNA synthetase (RS)/tRNA (25, 26) (Figure 1A). Acd’s small size and minimal linker reduce perturbations and heterogeneity, and its favorable photophysics support both steady-state and time-resolved measurements (27). Here, we incorporated Acd across all hH_v_1 structural domains and purified 12 of 14 constructs as stable, functional proteins. We then used Acd’s environmental sensitivity and FRET from tryptophan (Trp)/tyrosine (Tyr) to Acd to validate the protein folding and report Zn^2+^-dependent conformational changes. These data establish a platform for interrogating conformational heterogeneity and dynamics in the full-length hH_v_1.

## Results

### We generated a library of hH_v_1 constructs with Acd incorporated across the different structural domains.

Because available H_v_1 structural models were produced from either truncated or modified proteins (11, 12), we used an AlphaFold model of the full-length, dimeric hH_v_1 to guide Acd site selection (28, 29) (Figure 1B and Dataset S1). The model recapitulates the expected hH_v_1 architecture with high confidence: S0 as a short helix parallel to the membrane, S1-S4 transmembrane segments forming a canonical VSD, and a C-terminal helix extending from S4 that forms the CC intersubunit interface. In addition, two helices in the N-terminal domain (P1 and P2) were predicted (Figure 1C), albeit with low confidence. The dimer interface in the membrane is primarily formed by S4-S4 contacts (Figure S1A), although S1-S1 contacts have been observed experimentally (9, 13). This model appears to represent an intermediate state, with the first S4 positive charged residue R208 proximal to D112 in the selectivity filter, and the third S4 positive charged residue R211 proximal to F150 in the charge-transfer center (Figure S1B). Although the model’s accuracy and functional state are unknown, we used it as a rough starting point for selecting positions to incorporate Acd.

**Figure 2.**
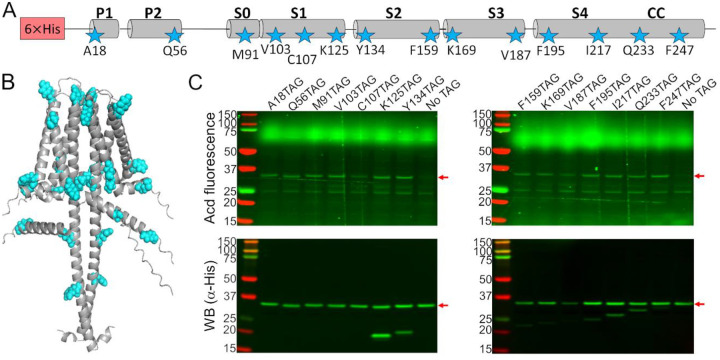
Acd was incorporated into 14 positions in the hH_v_1 sequence. (A) Cartoon showing the amino acids selected to be replaced by an amber stop codon in the hH_v_1 secondary structure to incorporate Acd (stars). (B) AlphaFold dimer hH_v_1 structural model with the amino acids selected to be replaced by Acd as cyan spheres. (C) Acd fluorescence gel (top) and Western blot (bottom) of cellular extracts after separation by SDS-PAGE showing the expression of the Acd-labeled hH_v_1 mutants. The No TAG lane contains an extract from cells grown under identical conditions (in the presence of Acd and the aminoacyl-tRNA synthetase/tRNA pair) and expressing hH_v_1 without an amber stop codon.

To test the feasibility and specificity of Acd incorporation in hH_v_1, we initially selected two positions in the intracellular domains: A18 in the N-terminal domain and F247 in the CC. For each position, the wild-type codon was replaced with an amber stop codon (TAG). Bacteria co-transformed with the Acd RS/tRNA pair and hH_v_1-TAG constructs expressed fluorescent full-length protein only when Acd was present in the culture medium (Figure 1D). We next generated a library of 14 constructs in which Acd was incorporated into every structural domain: the N-terminal domain, each helix of the VSD, and the CC (Figure 2A–B). Remarkably, most of the 14 hH_v_1-Acd proteins expressed at levels comparable to the control (No TAG) (Figure 2C and S2). As expected, several hH_v_1-Acd proteins also produced TAG-truncated products (Figure 2C). Increasing the Acd concentration in the *E. coli* growth medium did not reduce the fraction of truncated protein (Figure S3). One of the constructs, hH_v_1-C107Acd, showed a weak in-gel fluorescence signal, although the Western blot signal was comparable to that of other constructs.

### We successfully purified stable and functional hH_v_1 labeled with Acd at 12 of the 14 selected positions.

**Figure 3.**
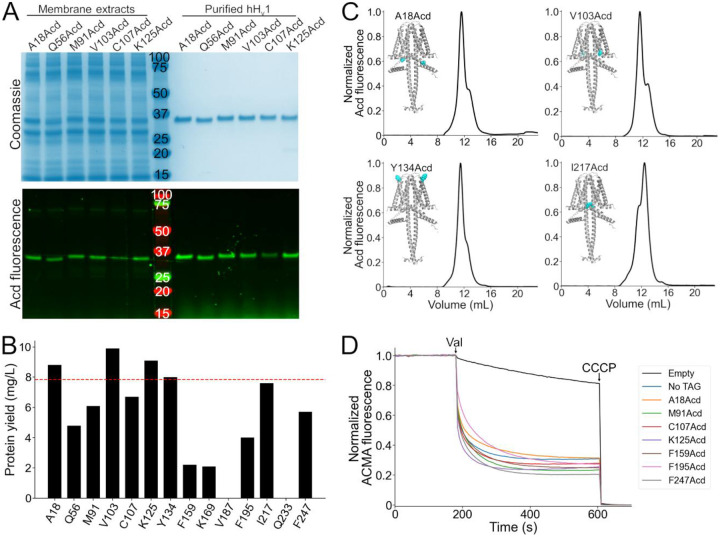
Purification and functional measurements of hH_v_1-Acd proteins. (A) Coomassie-stained (top) and Acd fluorescence (bottom) gels after separation by SDS-PAGE showing representative samples of the membrane extracts after solubilization (left) and the final purified protein after immobilized metal affinity chromatography (right). (B) Protein yield of the hH_v_1 protein with Acd replacing the amino acid at the indicated position. Note that V187Acd and Q233Acd contained no detectable protein after purification. The red line indicates the protein yield obtained from hH_v_1 without an amber stop codon in the presence of Acd and the aminoacyl-tRNA synthetase/tRNA pair (No TAG). (C) Representative fluorescence-detection size-exclusion chromatograms of the purified hH_v_1 proteins with Acd incorporated at the indicated amino acid position. Insets: AlphaFold dimer models, with the amino acid replaced by Acd shown as cyan spheres. (D) Representative liposome proton flux assay of asolectin proteoliposomes containing the indicated hH_v_1 protein. All purified proteins produced ACMA fluorescence quenching after the addition of valinomycin (Val). The protonophore CCCP was added at the end of the experiment as a control. The No TAG sample corresponds to proteoliposomes containing hH_v_1 without an amber stop codon expressed in the presence of Acd and the aminoacyl-tRNA synthetase/tRNA pair.

We attempted to purify all expressed hH_v_1-Acd constructs by immobilized metal affinity chromatography using the detergent Anzergent 3–12 (20, 21). For 12 of 14 constructs, purification yielded a single fluorescent band on SDS-PAGE (Figure 3A and S4). In contrast, no purified protein was detected for the V187Acd or Q233Acd constructs (Figure S4). Because hH_v_1-Q233Acd was robustly expressed (Figure 2C), its loss during purification suggests reduced stability. The hH_v_1-Acd yields were construct-dependent (Figure 3B), and the TAG-truncated products did not co-purify with the full-length protein (Figure 3A and S4).

We then evaluated the stability and homogeneity of the hH_v_1-Acd proteins using fluorescence-detection size exclusion chromatography (FSEC) (30). Similar to the control (Figure S5), most hH_v_1-Acd proteins showed a main peak (~11.5 mL) followed by a minor peak at higher elution volumes (Figure 3C and S6; Table S1). The proportion of these two peaks varied between constructs (Figure S6). To determine whether the purified hH_v_1-Acd proteins were functional, we reconstituted the FSEC fractions containing both peaks in asolectin liposomes and assessed their function using the liposome proton flux assay (20, 31). All 12 reconstituted constructs produced ACMA fluorescence quenching upon valinomycin addition, indicating that our purified proteins formed functional proton-permeable channels (Figure 3D and S7). These results highlight the versatility of GCE for incorporating Acd as a fluorescent probe for structural studies of hH_v_1.

### Acd was relatively insensitive to the hydrophobicity of its local environment.

**Figure 4.**
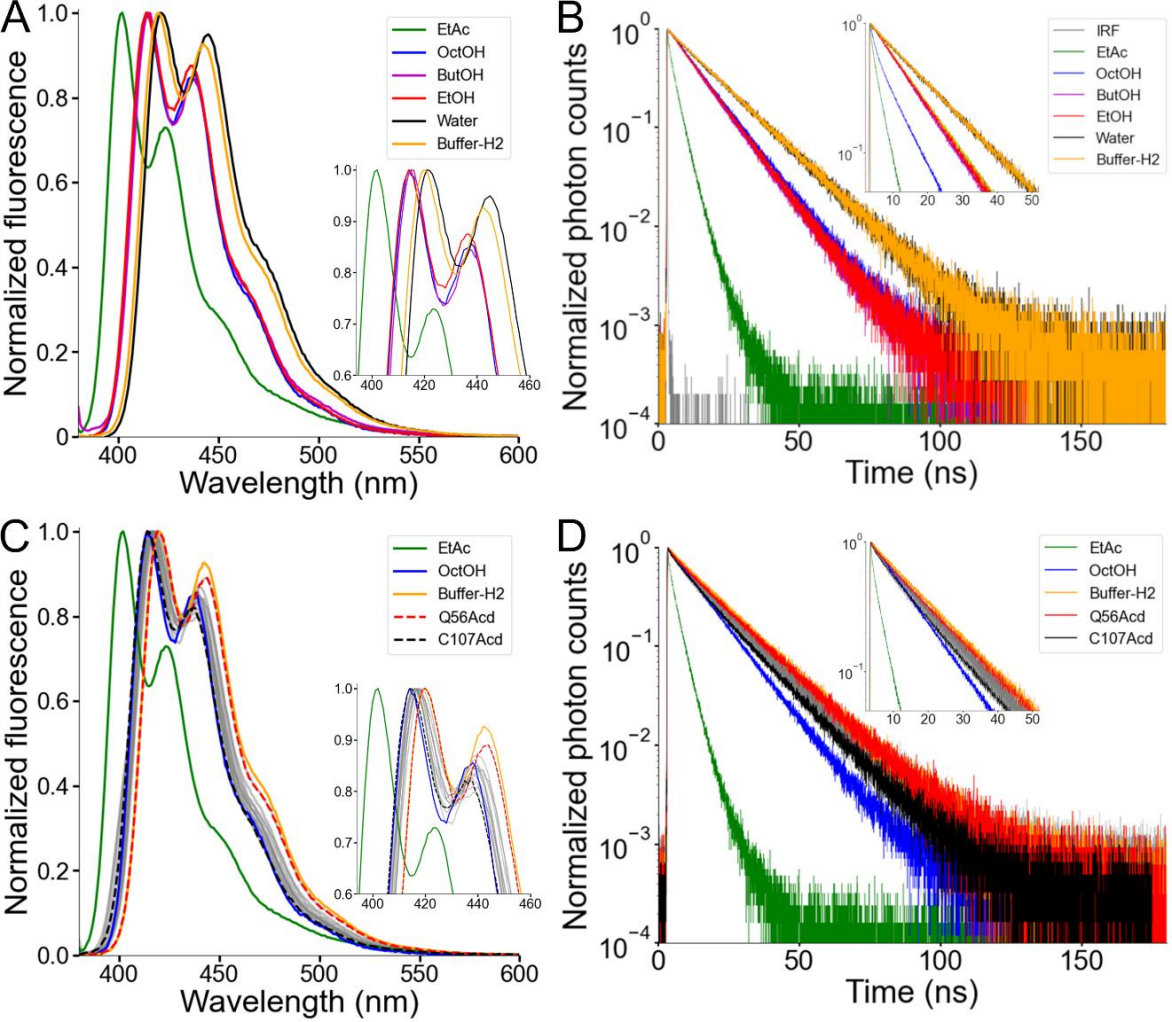
Acd showed a low environmental sensitivity in hH_v_1. (A) Normalized fluorescence emission spectra of the Acd amino acid dissolved in the indicated solvent (Excitation = 370 nm). EtAc: ethyl acetate, OctOH: 1-octanol, ButOH: 1-butanol, EtOH: ethanol. (B) Normalized fluorescence decay of the Acd amino acid dissolved in the indicated solvent. (C) Normalized fluorescence emission spectra of the hH_v_1-Acd proteins in Buffer-H2 (grey). The Acd amino acid spectra in selected solvents are also shown for reference. Q56Acd and C107Acd spectra are shown with broken lines. The emission maxima are listed in Table S3. (D) Normalized fluorescence decay of the hH_v_1-Acd proteins in Buffer-H2 (grey). The Acd amino acid decays in selected solvents are also shown for reference. Q56Acd and C107Acd decays are shown in red and black, respectively.

To explore how the local environment can alter Acd’s spectral properties, we measured the emission spectra of the free amino acid in different solvents (Figure 4A and Table S2). The environmental sensitivity of Acd has been studied previously (24, 27, 32), but we expanded this work here by using the aprotic solvent ethyl acetate (EtAc) and a series of alcohols with varying alkyl chain lengths. Consistent with a general solvent effect produced by polarity (16), the emission spectrum was blue-shifted by approximately 20 nm in EtAc, the least polar solvent measured, relative to water (Figure 4A and Table S2). Alcohols produced a consistently smaller blue shift, independent of their alkyl-chain length (Figure 4A). The spectrum in Buffer-H2, our standard experimental solution containing detergent micelles, was only very slightly blue-shifted relative to water (Figure 4A), suggesting that Acd did not substantially partition into the hydrophobic core of the detergent micelles. We also tested whether solvent polarity affects the fluorescence lifetime of Acd using time-correlated single photon counting (TCSPC). The fluorescence lifetime of Acd showed a similar trend to the spectral shifts (Figure 4B): EtAc shortened the lifetime markedly, Acd exhibited shorter lifetimes in alcohols than in water, and Acd in Buffer-H2 had a lifetime similar to that measured in water.

The spectral blue-shift and short lifetime of Acd in EtAc suggest that Acd may be able to report on the local environment at different sites in hH_v_1, especially those that are surface-exposed versus those that face the lipid core. The emission spectra of the hH_v_1-Acd proteins showed small, site-specific shifts (Figure 4C and Table S3). The local environment of Acd in hH_v_1 spanned the range between those observed for free Acd in Buffer-H2 and in alcohols, with Q56Acd in the N-terminal intracellular domain and C107Acd in S1 showing the most red-shifted and blue-shifted spectra, respectively (Figure 4C). Consistent with the spectra, the fluorescence lifetimes of the hH_v_1-Acd proteins fell between those measured for free Acd in Buffer-H2 and in alcohols, with Q56Acd exhibiting the slowest, and C107Acd the fastest, lifetimes (Figure 4D). The spectral properties of Acd in hH_v_1 agree with its location in the structural model, with C107 being the site closest to the detergent micelles’ hydrophobic core. The low sensitivity to polarity changes of Acd in different hH_v_1 local environments makes it well suited for measuring FRET (27, 33).

### FRET between Trp/Tyr and Acd in hH_v_1 suggested that the proteins are properly folded.

**Figure 5.**
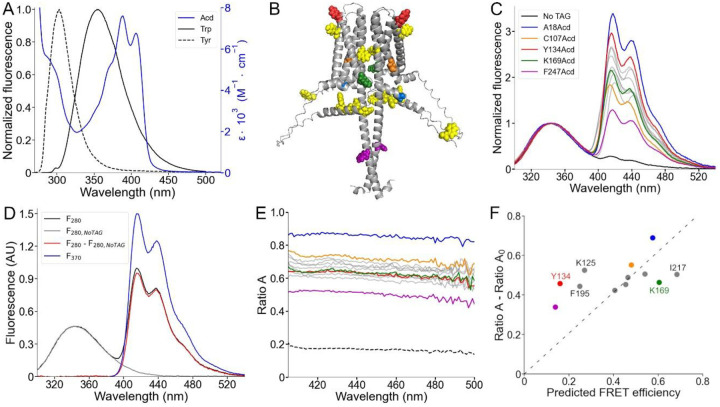
Spectral FRET analysis using Trp and Tyr donors and Acd as acceptor. (A) Fluorescence emission spectra of the intrinsic fluorescent amino acids Trp (black) and Tyr (black, broken lines), along with the Acd absorption spectrum in Buffer-H2 (blue). (B) AlphaFold dimer hH_v_1 structural model with the amino acids colored according to (C) and the native tryptophan and tyrosine residues shown as yellow spheres. (C) Normalized fluorescence spectra of the purified hH_v_1-Acd proteins (Excitation = 280 nm). The spectra were normalized by the Trp/Tyr fluorescence. The colors correspond to the indicated hH_v_1-Acd protein, and the remaining spectra are shown as grey traces in the figure. The No TAG sample (black) contains purified hH_v_1 protein without any amber stop codon expressed in the presence of Acd and the aminoacyl-tRNA synthetase/tRNA pair. (D) Example of the spectral FRET analysis procedure. The fluorescence spectrum of hH_v_1-Y134Acd obtained when exciting at 280 nm (black, *F*_*280*_) minus the normalized spectrum at the same excitation wavelength of the No TAG sample (grey, *F*_*280,NoTAG*_) produced the Acd emission spectrum shown in red (*F*_*280*_ – *F*_*280,NoTAG*_). *Ratio A* values were calculated by dividing this spectrum by the hH_v_1-Y134Acd fluorescence spectrum obtained by direct excitation at 370 nm (*F*_*370*_). (E) *Ratio A* traces of the hH_v_1-Acd proteins colored according to (C). The ratio between the spectra of free Acd amino acid excited at 280 and 370 nm (*Ratio A*_*0*_) is shown as broken lines. (F) Average *Ratio A* – *Ratio A*_*0*_ values (410–480 nm) as a function of the predicted FRET efficiencies from the AlphaFold hH_v_1 structural model, colored according to (C). The broken line is the best fit to the equation (*Ratio A* – *Ratio A*_*0*_) = m(Predicted FRET efficiency).

Trp and Tyr have fluorescence emission spectra that overlap with the Acd absorption spectrum and therefore can act as FRET donors to Acd, with relatively short *R*_*0*_ values (23.5 Å for Trp; 20.9 Å for Tyr) (22) (Figure 5A). Each hH_v_1 monomer contains four Trp and four Tyr residues (yellow spheres in Figure 5B). In this structural model, the Trp residues are located in the N-terminal domain (W4, W38, and W45) and midway along transmembrane segment S4 in the VSD (W207). The Tyr residues are located in the N-terminal domain (Y35 and Y42) and near the extracellular end of transmembrane segment S2 (Y134 and Y141).

To quantify Trp/Tyr to Acd FRET, we used spectral FRET analysis (34). The total emission spectrum from Trp/Tyr and Acd was collected (Figure 5C). The Acd emission spectrum was extracted by subtracting a scaled Trp/Tyr spectrum collected from control wild-type protein (*F*_*280,NoTAG*_). For each Acd-containing construct, the ratio of the extracted spectrum (*F*_*280*_) to the Acd spectrum with direct excitation (*F*_*370*_) was calculated as *Ratio A* (Figure 5D and S8). Because *Ratio A* is not wavelength-dependent, it reports the linearity of the detectors and the presence of significant contamination from other sources of fluorescence. The *Ratio A* component caused by the direct excitation of Acd (termed *Ratio A*_*0*_) was measured with free Acd (35) (Figure S9). The difference between *Ratio A* and *Ratio A*_*0*_ is directly proportional to the FRET efficiency.

As expected, the *Ratio A* values were largely flat across emission wavelengths for our hH_v_1-Acd proteins (Figure 5E). For all Acd sites, *Ratio A* > *Ratio A*_*0*_ (dashed line in Figure 5E), reflecting FRET between Trp/Tyr and Acd at every site in hH_v_1. Most *Ratio A* values were distributed between 0.60 and 0.80, with two clear exceptions: A18Acd (blue) and F247Acd (magenta) (Figure 5E). A18Acd showed the highest *Ratio A*, consistent with the multiple Trp/Tyr residues in the N-terminal domain. Conversely, F247Acd showed the lowest *Ratio A*, consistent with its location in the CC, far from most Trp/Tyr residues (Figure 5B).

We next compared *Ratio A – Ratio A*_*0*_ values with FRET efficiencies predicted by the AlphaFold structural model. We modeled rotamer ensembles for Trp, Tyr, and our Acd sites in the hH_v_1 structural model using chiLife (36) and calculated donor-acceptor distance distributions. These distributions were then used to calculate FRET efficiencies (details in SI Text). The experimentally determined *Ratio A – Ratio A*_*0*_ values showed a moderate correlation (Pearson’s r = 0.54) with the calculated efficiencies (Figure 5F).

There were two spatially clustered deviations between experiment and model. Positions at the extracellular ends of the VSD helices (Y134 in S2, K125 in S1, and F195 in S4) showed higher experimental FRET than predicted, indicating shorter effective donor-acceptor distances relative to the model. Conversely, positions on the intracellular side of the VSD (K169 in S3 and I217 in S4) showed lower experimental FRET than predicted, implying longer effective donor-acceptor distances. Together, the spatial clustering of residues with similar deviations indicates specific deficits in the AlphaFold model and can guide future improvements.

### Zinc binding changed the conformation of hH_v_1.

**Figure 6.**
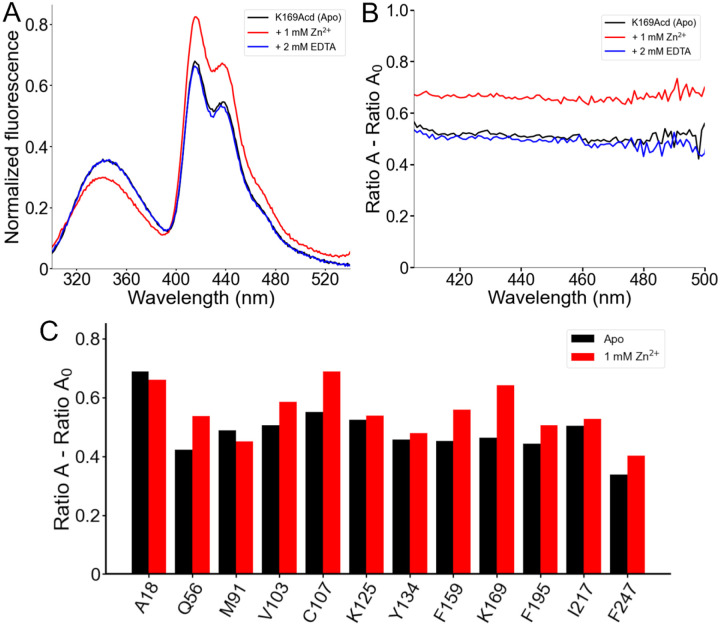
FRET between Trp/Tyr and Acd reports a conformational rearrangement in response to Zn^2+^. (A) Normalized fluorescence emission spectra of hH_v_1-K169Acd (Excitation = 280 nm) in Buffer-H2 in the absence of Zn^2+^ (black, Apo), in the presence of 1 mM Zn^2+^ (red), or in the presence of 1 mM Zn^2+^ and 2 mM EDTA (blue). Spectra were normalized by the maximum intensity of the Acd emission spectrum of the same sample excited at 370 nm. (B) Spectral FRET analysis of hH_v_1-K169Acd in Buffer-H2 in the absence of Zn^2+^ (black, Apo), in the presence of 1 mM Zn^2+^ (red), or in the presence of 1 mM Zn^2+^ and 2 mM EDTA (blue). (C) Spectral FRET analysis of the hH_v_1-Acd proteins in Buffer-H2 in the absence (black, Apo) or presence of 1 mM Zn^2+^ (red).

We next asked whether FRET from Trp/Tyr to Acd could report conformational changes upon addition of Zn^2+^, the classical inhibitor of H_v_1 proton currents in cells (7). The two experimental structures of H_v_1 were resolved with this cation bound to the extracellular side of the VSD (11, 12). We observed site-specific spectral changes in the hH_v_1-Acd proteins in the presence of Zn^2+^ (Figure 6 and S10). The largest changes occurred for hH_v_1-K169Acd, where 1 mM Zn^2+^ caused a decrease in the Trp/Tyr emission and an increase in the Acd emission (Figure 6A). This decrease in donor fluorescence, paired with increased acceptor fluorescence, is the hallmark of increased FRET and was corroborated by spectral analysis (Figure 6B). Additionally, a slight blue shift of the Trp/Tyr fluorescence was observed for all hH_v_1-Acd proteins in the presence of Zn^2+^ (Figure S11). These changes were completely reversed by the addition of EDTA, confirming that the changes arise from a reversible conformational change upon Zn^2+^ binding (Figure 6A and 6B). Large changes in effective FRET efficiency were observed with Acd incorporated at Q56 in the N-terminal domain and C107, F159, and K169 in the VSD (Figure 6C). Notably, these positions are intracellular, even though H_v_1 currents are inhibited by extracellular Zn^2+^ (7, 37). These data demonstrate that Zn^2+^ binds to hH_v_1 and alters its conformation in detergent micelles, consistent with a nuclear magnetic resonance study of the VSD of hH_v_1 (12).

## Discussion

Incorporating Acd into hH_v_1 by GCE opens new possibilities for studying the structure and function of this channel. We systematically incorporated Acd at multiple positions in hH_v_1 and confirmed protein stability and function after purification. 12 of 14 functional hH_v_1-Acd proteins produced acceptable yields upon purification, despite the additional challenges associated with recombinant expression of a human membrane protein in *E. coli*. Two factors likely underlie the robustness of our system: (i) an optimized purification protocol for hH_v_1 (20, 21) that offsets the expected reduction in yield associated with GCE (22, 25), and (ii) the high efficiency of the evolved aminoacyl-tRNA synthetase for incorporating Acd (17, 27).

Fluorescence spectroscopy has been used to detect conformational changes in H_v_1. Sites at the extracellular portion of S4 (38) and S1 (39) in the VSD of *Ciona intestinalis* H_v_1 have been labeled with the fluorophores Alexa488 and tetramethylrhodamine (TAMRA) through cysteine-selective reagents to capture conformational changes during activation using voltage-clamp fluorometry. Similarly, the fluorescent noncanonical amino acid 3-(6-acetylnaphthalen-2-ylamino)-2-aminopropanoic acid (Anap) has been incorporated at several sites in the S4 in hH_v_1 (40) to study conformational changes in response to voltage and pH. In these cases, the fluorophore’s environmental sensitivity has been used as a readout of conformational changes. By contrast, FRET provides richer structural information due to its distance dependence. Single-molecule FRET has been measured in purified hH_v_1 labeled with Cy3 and Cy5 to track the VSD movements in response to voltage, pH, cholesterol, and zinc (14, 41). The small size and minimal linker of Acd, along with its low environmental sensitivity and high photostability, are advantageous for measuring FRET in hH_v_1 compared with previously used fluorophores.

The high number of FRET donors, 4 tryptophans and 4 tyrosines per subunit, limits the utility of the Trp/Tyr-Acd FRET pair as a strategy to measure conformational rearrangements in hH_v_1. Our library of a dozen single Acd-incorporating constructs, with sites distributed across the entire primary sequence of the protein, opens the door to using Acd as a FRET donor. We have previously used time-resolved transition metal ion FRET (tmFRET), which utilizes a transition metal ion as a FRET acceptor (27, 33), to measure conformational energetics in the bacterial cyclic nucleotide-gated ion channel SthK (42). However, in that work, we incorporated Acd at a single position in the intracellular C-terminal domain of SthK. With Acd incorporated at multiple sites in full-length hH_v_1, we can interrogate its gating mechanisms in unprecedented detail.

## Materials and Methods

### Protein expression and purification.

hH_v_1 was expressed and purified according to the published protocol with minor modifications (20, 21). Briefly, fresh competent BL21-Gold(DE3) cells (Agilent) were co-transformed with the His-EK-hH_v_1-C107A-C249A.pET15-b (hH_v_1-Cysless, referred to as No TAG) and MjA9Acd-RS.pDule2 (22, 23) plasmids (sequences in SI Text) using the heat shock method. For expressing hH_v_1-Acd proteins, the codon corresponding to the selected position was changed to the amber stop codon (TAG) by site-directed mutagenesis. All plasmids were sequenced before use. Pre-cultures were grown overnight in LB medium supplemented with 0.2% glucose, 0.4 mg/mL ampicillin, and 0.1 mg/mL spectinomycin at 37 °C and 250 rpm. The next day, cultures were diluted 1:100 in complete autoinduction medium (43) supplemented with 0.4 mg/mL ampicillin and 0.1 mg/mL spectinomycin, and then grown at 37 °C and 250 rpm until an OD_600_ of 1.5 was reached. The protein expression level was proportional to the Acd concentration in the culture medium (Figure S11). We chose 0.6 mM Acd in the culture medium as optimal for hH_v_1 expression, as higher concentrations only slightly increased the final biomass (Figure S11B). Cultures supplemented with 0.6 mM Acd were grown overnight at 20 °C and 250 rpm. Cells were then harvested, resuspended in Buffer-H1 (50 mM Tris, 150 mM NaCl, 1 mM benzamidine, 0.17 mg/mL PMSF, pH 8.0) supplemented with 0.5 mg/mL lysozyme, and incubated for 30 min at 4 °C before being flash-frozen. After thawing, the extracts were supplemented with 5 mM MgCl_2_, fresh protease inhibitors, and 12.5 μg/mL DNase I, incubated for 1 h at 4 °C, sonicated, and centrifuged at 100,000g at 4 °C for 1 h. The membrane pellet was resuspended in Buffer-H1 and stored at −80 °C until further use. After thawing, the membranes were solubilized with 1.5% Anzergent 3–12 (Anatrace) (Anz3-12) at room temperature for 1 hour. Insoluble material was removed by centrifuging at 100,000g and 4 °C for 1 h, and the solubilized membrane extract was incubated with His-Pur Ni-NTA (Thermo Scientific) resin equilibrated with Buffer-H2 (50 mM Tris, 150 mM NaCl, 12 mM Anz3-12, pH 8.0) for 1 hour at room temperature. Resin was collected in a gravity column and washed with 20 column volumes (CV) of Buffer-H2, followed by 16 CV of Buffer-H2 with 90 mM imidazole. The hH_v_1 protein was eluted with 20 CV of Buffer-H2 with 0.5 M imidazole, was concentrated with 50kDa cut-off centrifugal filters, and imidazole was removed by FSEC (Ex/Em = 385/450 nm) in an ENrich SEC 650 (Bio-Rad) column with Buffer-H2. The hH_v_1-containing fractions were concentrated to 0.5–1.5 mg/mL, depending on the amount of purified protein, and then aliquoted, flash-frozen, and stored at −80 °C until use.

### Reconstitution and fluorescence proton flux assays.

The hH_v_1-Acd samples were reconstituted and assayed using a liposome proton flux assay, as described previously, with minor modifications (20). Briefly, soy polar lipid extract (Avati) in chloroform was dried overnight at room temperature in a rotary evaporator under vacuum and then hydrated in Buffer-K (20 mM HEPES, 150 mM KCl, and 1 mM EDTA, pH 7.0) to a concentration of 10 mg/mL. The vesicles were sonicated in a water bath until the solution became translucent, aliquoted, flash-frozen, and stored at −80 °C until further use. Reconstitutions were performed at a 1:100 (protein:lipid, by mass) ratio by mixing 12.5 μg of purified hH_v_1-Acd with 1.25 mg of liposomes in a final volume of 330 μL Buffer-K containing 8 mM Anz3-12. The mixture was incubated at room temperature for 1 h, then diluted with 5 mL of Buffer-K and incubated for 30 min. The mixture was further incubated with three cumulative pulses of 100 mg Bio-Beads SM2 (Bio-Rad), each for 1 h at room temperature. After the final Bio-Beads pulse, the mixture was incubated overnight at 4 °C. The next day, two successive 100 mg additions of Bio-Beads were performed, with a 1-hour incubation at room temperature following each addition. Bio-Beads were removed in a gravity column, the mixture was diluted with 10 mL of Buffer-K, and then centrifuged at 150,000g for 2 hours at 4 °C. The proteoliposome pellets were resuspended in 250 μL of Buffer-K, aliquoted, flash-frozen, and stored at −80 °C until further use. On the day of the liposome proton flux assays, samples were thawed at 37 °C and then placed on ice. The sample (40 μL) was diluted in Buffer-Na (20 mM HEPES, 150 mM NaCl, 1 mM EDTA, pH 7.0). 9-Amino-6-chloro-2-methoxyacridine (ACMA; Sigma) was added to a final concentration of 2 μM from a 2 mM stock solution in DMSO, and the mixture was incubated at room temperature for 5 minutes before the fluorescence measurement began. The baseline was recorded for 3 min (*F*_*max*_). Subsequently, 10 nM of valinomycin (Cell Signaling) was added from a 10 μM stock solution in DMSO, followed by 1 μM carbonyl cyanide m-chlorophenyl hydrazone (CCCP; Sigma) from a 1 mM stock solution in DMSO to record *F*_*min*_. The final volume was 2 mL. The fluorescence signal (*F(t)*) was normalized according to the equation (*F(t)*-*F*_*min*_)/(*F*_*max*_-*F*_*min*_). Fluorescence measurements were performed at 5-second intervals with a 2-second integration time using a Fluorolog-3 spectrometer (Horiba) configured to an excitation wavelength of 410 nm (5 nm bandpass) and an emission wavelength of 490 nm (1 nm bandpass) at room temperature.

### Absorption and fluorescence spectra recordings.

The absorption and fluorescence spectra were recorded in the indicated solvents or fresh Buffer-H2 at room temperature. Absorption spectra were recorded in a DU 800 Spectrometer (Beckman Coulter) with a wavelength interval of 0.5 nm and a scan speed of 600 nm/min. Fluorescence emission spectra were measured using a Fluorolog-3 spectrometer (Horiba) with an integration time of 0.1 s, 1 nm increments, and excitation and emission slits of 5 nm. For solubilizing Acd in different solvents, a saturated solution of the amino acid was prepared by dissolving 0.5 mg of Acd powder in the respective solvent. Acd spectra were corrected by subtracting a blank of the solvent. hH_v_1-Acd proteins were measured at concentrations ranging from 180 to 240 nM in Buffer-H2. Each spectrum was corrected by subtracting a blank with the solvent or Buffer-H2 before adding the protein sample to the cuvette.

### Spectral FRET analysis.

We used our previously established spectral FRET analysis to remove contamination caused by direct excitation of Acd by 280 nm light (35). This method had the added benefit of eliminating errors arising from the recording system’s transfer function, variations in the acceptor’s quantum yield, or variations in the total concentration of fluorescent molecules. A Trp/Tyr spectrum was collected from control protein without any TAG codons (no Acd incorporation). This was used to subtract the Trp/Tyr emission spectra collected at 280 nm for each Acd-incorporating protein. This yielded the extracted Acd spectrum, *F*_*280*_, that had two components: the component due to direct excitation of Acd, $F_{280}^{\text{direct}}$, and the component due to FRET, $F_{280}^{\text{FRET}}$. *F*_*280*_ was normalized by the total Acd emission with 370 nm excitation, *F*_*370*_. The resulting ratio, termed *Ratio A*, can be expressed as: $\text{Ratio A}=\frac{F_{280}}{F_{370}}=\frac{F_{280}^{\text{direct}}}{F_{370}}+\frac{F_{280}^{\text{FRET}}}{F_{370}}$. The direct excitation component, $\frac{F_{280}^{\text{direct}}}{F_{370}}$, termed *Ratio A*_*0*_, was measured using free Acd amino acid in Buffer-H2. We then quantified the relative FRET efficiency as $\text{Ratio A}-{\text{Ratio A}}_{0}=\frac{F_{280}^{\text{FRET}}}{F_{370}}$. This quantity is directly proportional to FRET efficiency. An average *Ratio A* value was calculated between 410 and 480 nm.

### Time-resolved fluorescence measurements.

TCSPC measurements were performed in a FluoTime 300 spectrometer equipped with a PMA Hybrid 40 detector and LDH-P-C-375 laser diode head (PicoQuant) at room temperature. Emitted photons were detected at 446 nm with the emission polarizer at the magic angle. The TCSPC measurements were performed in the indicated solvent or fresh Buffer-H2. The hH_v_1-Acd samples were measured at a concentration of 1 μM and the Acd amino acid at 500 nM. The count rate was always less than 1% of the excitation repetition rate to avoid pile-up distortions of the fluorescence decay. The IRF was measured using a dilute Ludox solution in water.

## Supplementary Material

Supplement 1

## References

[1] Sasaki M., Takagi M., Okamura Y., A Voltage Sensor-Domain Protein Is a Voltage-Gated Proton Channel. Science 312, 589–592 (2006). 16556803 10.1126/science.1122352

[2] Ramsey I. S., Moran M. M., Chong J. A., Clapham D. E., A voltage-gated proton-selective channel lacking the pore domain. Nature 440, 1213–1216 (2006). 16554753 10.1038/nature04700 PMC4084761

[3] Cherny V. V., Markin V. S., DeCoursey T. E., The voltage-activated hydrogen ion conductance in rat alveolar epithelial cells is determined by the pH gradient. The Journal of General Physiology 105, 861–896 (1995). 7561747 10.1085/jgp.105.6.861 PMC2216954

[4] Carmona E. M., , Gating charge displacement in a monomeric voltage-gated proton (H_v_1) channel. Proc. Natl. Acad. Sci. U.S.A. 115, 9240–9245 (2018). 30127012 10.1073/pnas.1809705115 PMC6140481

[5] Carmona E. M., , The voltage sensor is responsible for ΔpH dependence in H_v_1 channels. Proc. Natl. Acad. Sci. U.S.A. 118, e2025556118 (2021). 33941706 10.1073/pnas.2025556118 PMC8126849

[6] Pathak M. M., , The Hv1 proton channel responds to mechanical stimuli. Journal of General Physiology 148, 405–418 (2016). 27799320 10.1085/jgp.201611672 PMC5089936

[7] Cherny V. V., DeCoursey T. E., pH-Dependent Inhibition of Voltage-Gated H^+^ Currents in Rat Alveolar Epithelial Cells by Zn^2+^ and Other Divalent Cations. The Journal of General Physiology 114, 819–838 (1999). 10578017 10.1085/jgp.114.6.819 PMC2230650

[8] DeCoursey T. E., Cherny V. V., Voltage-activated hydrogen ion currents. J. Membarin Biol. 141 (1994). 10.1007/BF00235130 7528804

[9] Lee S.-Y., Letts J. A., MacKinnon R., Dimeric subunit stoichiometry of the human voltage-dependent proton channel H_v_1. Proc. Natl. Acad. Sci. U.S.A. 105, 7692–7695 (2008). 18509058 10.1073/pnas.0803277105 PMC2409406

[10] Koch H. P., , Multimeric nature of voltage-gated proton channels. Proc. Natl. Acad. Sci. U.S.A. 105, 9111–9116 (2008). 18583477 10.1073/pnas.0801553105 PMC2449373

[11] Takeshita K., , X-ray crystal structure of voltage-gated proton channel. Nat Struct Mol Biol 21, 352–357 (2014). 24584463 10.1038/nsmb.2783

[12] Bayrhuber M., , Nuclear Magnetic Resonance Solution Structure and Functional Behavior of the Human Proton Channel. Biochemistry 58, 4017–4027 (2019). 31365236 10.1021/acs.biochem.9b00471

[13] Li Q., , Resting state of the human proton channel dimer in a lipid bilayer. Proc. Natl. Acad. Sci. U.S.A. 112 (2015). 10.1073/pnas.1515043112 PMC4640771 26443860

[14] Han S., , Structural dynamics determine voltage and pH gating in human voltage-gated proton channel. eLife 11, e73093 (2022). 35244539 10.7554/eLife.73093 PMC8926398

[15] Henzler-Wildman K., Kern D., Dynamic personalities of proteins. Nature 450, 964–972 (2007). 18075575 10.1038/nature06522

[16] Lakowicz J. R., Ed., Principles of Fluorescence Spectroscopy (Springer US, 2006).

[17] Gordon S. E., Munari M., Zagotta W. N., Visualizing conformational dynamics of proteins in solution and at the cell membrane. eLife 7, e37248 (2018). 29923827 10.7554/eLife.37248 PMC6056233

[18] Zagotta W. N., , Measuring conformational equilibria in allosteric proteins with time-resolved tmFRET. Biophysical Journal 123, 2050–2062 (2024). 38303511 10.1016/j.bpj.2024.01.033 PMC11309986

[19] Taraska J. W., Puljung M. C., Zagotta W. N., Short-distance probes for protein backbone structure based on energy transfer between bimane and transition metal ions. Proc. Natl. Acad. Sci. U.S.A. 106, 16227–16232 (2009). 19805285 10.1073/pnas.0905207106 PMC2741476

[20] Carmona E. M., Cortes D. M., Cuello L. G., A novel method for expressing and purifying large quantities of functional and stable human voltage-gated proton channel (HH_V_1). Protein Science 34, e70017 (2025). 39865375 10.1002/pro.70017 PMC11761714

[21] Carmona E., Cortes M., Cuello L., Expression and Purification of the Human Voltage-Gated Proton Channel (hH_v_1). BIO-PROTOCOL 15 (2025). 10.21769/BioProtoc.5344 PMC12254590 40657542

[22] Speight L. C., , Efficient Synthesis and In Vivo Incorporation of Acridon-2-ylalanine, a Fluorescent Amino Acid for Lifetime and Förster Resonance Energy Transfer/Luminescence Resonance Energy Transfer Studies. J. Am. Chem. Soc. 135, 18806–18814 (2013). 24303933 10.1021/ja403247j PMC4041393

[23] Sungwienwong I., , Improving target amino acid selectivity in a permissive aminoacyl tRNA synthetase through counter-selection. Org. Biomol. Chem. 15, 3603–3610 (2017). 28397914 10.1039/c7ob00582b PMC5507695

[24] Jones C. M., , Genetic encoding of a highly photostable, long lifetime fluorescent amino acid for imaging in mammalian cells. Chem. Sci. 12, 11955–11964 (2021). 34976337 10.1039/d1sc01914g PMC8634729

[25] Liu C. C., Schultz P. G., Adding New Chemistries to the Genetic Code. Annu. Rev. Biochem. 79, 413–444 (2010). 20307192 10.1146/annurev.biochem.052308.105824

[26] Young T. S., Ahmad I., Yin J. A., Schultz P. G., An Enhanced System for Unnatural Amino Acid Mutagenesis in E. coli. Journal of Molecular Biology 395, 361–374 (2010). 19852970 10.1016/j.jmb.2009.10.030

[27] Zagotta W. N., , An improved fluorescent noncanonical amino acid for measuring conformational distributions using time-resolved transition metal ion FRET. eLife 10, e70236 (2021). 34623258 10.7554/eLife.70236 PMC8500717

[28] Jumper J., , Highly accurate protein structure prediction with AlphaFold. Nature 596, 583–589 (2021). 34265844 10.1038/s41586-021-03819-2 PMC8371605

[29] Kim G., , Easy and accurate protein structure prediction using ColabFold. Nat Protoc 20, 620–642 (2025). 39402428 10.1038/s41596-024-01060-5

[30] Kawate T., Gouaux E., Fluorescence-Detection Size-Exclusion Chromatography for Precrystallization Screening of Integral Membrane Proteins. Structure 14, 673–681 (2006). 16615909 10.1016/j.str.2006.01.013

[31] Lee S.-Y., Letts J. A., MacKinnon R., Functional Reconstitution of Purified Human H_v_1 H^+^ Channels. Journal of Molecular Biology 387, 1055–1060 (2009). 19233200 10.1016/j.jmb.2009.02.034 PMC2778278

[32] Szymańska A., Wegner K., Łankiewicz L., Synthesis of *N*-[(*tert*-Butoxy)carbonyl]-3-(9,10-dihydro-9-oxoacridin-2-yl)-L-alanine, a New Fluorescent Amino Acid Derivative. Helvetica Chimica Acta 86, 3326–3331 (2003).

[33] Gordon S. E., , Long-distance tmFRET using bipyridyl- and phenanthroline-based ligands. Biophysical Journal 123, 2063–2075 (2024). 38350449 10.1016/j.bpj.2024.01.034 PMC11309967

[34] Clegg R. M., “[18] Fluorescence resonance energy transfer and nucleic acids” in Methods in Enzymology, (Elsevier, 1992), pp. 353–388. 10.1016/0076-6879(92)11020-j 1406315

[35] Zheng J., Varnum M. D., Zagotta W. N., Disruption of an Intersubunit Interaction Underlies Ca^2+^-Calmodulin Modulation of Cyclic Nucleotide-Gated Channels. J. Neurosci. 23, 8167–8175 (2003). 12954880 10.1523/JNEUROSCI.23-22-08167.2003 PMC6740483

[36] Tessmer M. H., Stoll S., chiLife: An open-source Python package for in silico spin labeling and integrative protein modeling. PLoS Comput Biol 19, e1010834 (2023). 37000838 10.1371/journal.pcbi.1010834 PMC10096462

[37] Qiu F., , Molecular mechanism of Zn^2+^ inhibition of a voltage-gated proton channel. Proc. Natl. Acad. Sci. U.S.A. 113 (2016). 10.1073/pnas.1604082113 PMC5056077 27647906

[38] Gonzalez C., Koch H. P., Drum B. M., Larsson H. P., Strong cooperativity between subunits in voltage-gated proton channels. Nat Struct Mol Biol 17, 51–56 (2010). 20023639 10.1038/nsmb.1739 PMC2935852

[39] Mony L., Berger T. K., Isacoff E. Y., A specialized molecular motion opens the Hv1 voltage-gated proton channel. Nat Struct Mol Biol 22, 283–290 (2015). 25730777 10.1038/nsmb.2978 PMC4385474

[40] Suárez-Delgado E., Orozco-Contreras M., Rangel-Yescas G. E., Islas L. D., Activation-pathway transitions in human voltage-gated proton channels revealed by a noncanonical fluorescent amino acid. eLife 12, e85836 (2023). 36695566 10.7554/eLife.85836 PMC9925047

[41] Han S., , Cholesterol inhibits human voltage-gated proton channel hHv1. Proc. Natl. Acad. Sci. U.S.A. 119, e2205420119 (2022). 36037383 10.1073/pnas.2205420119 PMC9457259

[42] Eggan P., Gordon S. E., Zagotta W. N., Domain Coupling in Allosteric Regulation of SthK Measured Using Time-Resolved Transition Metal Ion FRET. [Preprint] (2025). Available at: https://elifesciences.org/reviewed-preprints/106892v2 [Accessed 2 August 2025]. 10.7554/eLife.106892 PMC12342821 40792615

[43] Studier F. W., Protein production by auto-induction in high-density shaking cultures. Protein Expression and Purification 41, 207–234 (2005). 15915565 10.1016/j.pep.2005.01.016

