# Supplementary material for: Interrogating the structure and function of the human voltage-gated proton channel (hHv1) with a fluorescent noncanonical amino acid
Source: bioRxiv. 2025 Dec 4:2025.10.23.684235. Originally published 2025 Oct 24. Preprint. [Version 2] doi: 10.1101/2025.10.23.684235 (PMC12633253; doi:10.1101/2025.10.23.684235)
Supplement: Supplement 1 [file NIHPP2025.10.23.684235v2-supplement-1.pdf]

bioRxiv preprint doi: https://doi.org/10.1101/2025.10.23.684235; this version posted December 4, 2025. The copyright holder for this preprint (which was not certified by peer review) is the author/funder, who has granted bioRxiv a license to display the preprint in perpetuity. It is made available under aCC-BY 4.0 International license.

Supplementary Text

Calculation of the FRET efficiency from the structural model. We calculated the FRET efficiency using the AlphaFold model of the full-length hH_v1 (Dataset S1). For each Acd position, the measured steady-state FRET would correspond to the mean FRET efficiency (E_{FRET}), which will be proportional to the *Ratio A – Ratio A₀* value obtained from the spectral FRET analysis.

For *n* number of donors and two acceptors in the hH_v1 dimer:

$$E_{FRET} = \sum_{i=1}^{n} P_{Ex,i} E_i \qquad (1)$$

where $P_{Ex,i}$ is the probability of excitation and E_i is the FRET efficiency of the *i*th donor. $P_{Ex,i}$ is the number of photons absorbed by the *i*th donor divided by the total number of photons absorbed by the total number *n* of donors, which is quantified by the donor's extinction coefficient. Supposing the same extinction coefficient for each of the Trp (e_W = 5,501 M^{-1} cm^{-1}) and Tyr (e_Y = 1,209 M^{-1} cm^{-1})(1), equation 1 can be written as:

$$E_{FRET} = \sum_{i=1}^{n} \left(\frac{\varepsilon_i}{\sum_{i=1}^{n} \varepsilon_i} \right) E_i = \frac{1}{n_W \varepsilon_W + n_Y \varepsilon_Y} \left(\varepsilon_W \sum_{i=1}^{n_W} E_{W,i} + \varepsilon_Y \sum_{i=1}^{n_Y} E_{Y,i} \right) \qquad (2)$$

where the sum of the total number of Trp (n_W) and Tyr (n_Y) is equal to *n*. The efficiency of FRET for the *i*th donor can be expressed as the sum of rates of energy transfer to each of the *j* = 2 Acd acceptors, k_{ij}, divided by the total rates of photon emission. Therefore, the right terms of equation 2 can be expressed as:

$$\sum_{i=1}^{n_W} E_{W,i} = \sum_{i=1}^{n_W} \left(\frac{\sum_{j=1}^{2} k_{ij}}{\sum_{j=1}^{2} k_{ij} + (1/\tau_W)} \right) \quad \text{and} \quad \sum_{i=1}^{n_Y} E_{Y,i} = \sum_{i=1}^{n_Y} \left(\frac{\sum_{j=1}^{2} k_{ij}}{\sum_{j=1}^{2} k_{ij} + (1/\tau_Y)} \right) \qquad (3)$$

where the Trp lifetime (t_W = 3.1 ns) and tyrosine lifetime (t_Y = 3.6 ns) in the absence of donors are considered constant(1). Finally, each energy transfer rate k_{ij} is a function of the distance r_{ij} between the *i*th donor and the *j*th acceptor:

$$k_{ij}(r) = \frac{1}{\tau_D} \left(\frac{R_0}{r_{ij}} \right)^6 \qquad (4)$$

where the donor lifetime t_D and R_0 would be t_W and 23.5 Å or t_Y and 20.9 Å for Trp or Tyr residues, respectively. Calculating E_{FRET} using the above equations considers a fixed distance between donors and acceptors. A better approach is to consider the distance distributions $P_{ij}(r)$ produced by the different rotameric

bioRxiv preprint doi: https://doi.org/10.1101/2025.10.23.684235; this version posted December 4, 2025. The copyright holder for this preprint (which was not certified by peer review) is the author/funder, who has granted bioRxiv a license to display the preprint in perpetuity. It is made available under aCC-BY 4.0 International license.

states of the FRET pair, which can be modeled easily using chiLife(2). Then, equation 4 will be replaced by equation 5:

$$k_{ij}(r) = \int_0^\infty P_{ij}(r)\left[\frac{1}{\tau_D}\left(\frac{R_0}{r}\right)^6\right]dr \qquad (5).$$

DNA plasmid sequences in FASTA format.

>His-EK-hHv1-C107A-C249A.pET15-b
TTGAGATCCTTTTTTTCTGCGCGTAATCTGCTGCTTGCAAACAAAAAAACCAC
CGCTACCAGCGGTGGTTTGTTTGCCGGATCAAGAGCTACCAACTCTTTTTCC
GAAGGTAACTGGCTTCAGCAGAGCGCAGATACCAAATACTGTCCTTCTAGT
GTAGCCGTAGTTAGGCCACCACTTCAAGAACTCTGTAGCACCGCCTACATA
CCTCGCTCTGCTAATCCTGTTACCAGTGGCTGCTGCCAGTGGCGATAAGTC
GTGTCTTACCGGGTTGGACTCAAGACGATAGTTACCGGATAAGGCGCAGCG
GTCGGGCTGAACGGGGGGTTCGTGCACACAGCCCAGCTTGGAGCGAACGA
CCTACACCGAACTGAGATACCTACAGCGTGAGCTATGAGAAAGCGCCACGC
TTCCCGAAGGGAGAAAGGCGGACAGGTATCCGGTAAGCGGCAGGGTCGGA
ACAGGAGAGCGCACGAGGGAGCTTCCAGGGGGAAACGCCTGGTATCTTTA
TAGTCCTGTCGGGTTTCGCCACCTCTGACTTGAGCGTCGATTTTTGTGATGC
TCGTCAGGGGGGCGGAGCCTATGGAAAAACGCCAGCAACGCGGCCTTTTT
ACGGTTCCTGGCCTTTTGCTGGCCTTTTGCTCACATGTTCTTTCCTGCGTTA
TCCCCTGATTCTGTGGATAACCGTATTACCGCCTTTGAGTGAGCTGATACCG
CTCGCCGCAGCCGAACGACCGAGCGCAGCGAGTCAGTGAGCGAGGAAGC
GGAAGAGCGCCTGATGCGGTATTTTCTCCTTACGCATCTGTGCGGTATTTCA
CACCGCATATATGGTGCACTCTCAGTACAATCTGCTCTGATGCCGCATAGTT
AAGCCAGTATACACTCCGCTATCGCTACGTGACTGGGTCATGGCTGCGCCC
CGACACCCGCCAACACCCGCTGACGCGCCCTGACGGGCTTGTCTGCTCCC
GGCATCCGCTTACAGACAAGCTGTGACCGTCTCCGGGAGCTGCATGTGTCA
GAGGTTTTCACCGTCATCACCGAAACGCGCGAGGCAGCTGCGGTAAAGCT
CATCAGCGTGGTCGTGAAGCGATTCACAGATGTCTGCCTGTTCATCCGCGT
CCAGCTCGTTGAGTTTCTCCAGAAGCGTTAATGTCTGGCTTCTGATAAAGCG
GGCCATGTTAAGGGCGGTTTTTTCCTGTTTGGTCACTGATGCCTCCGTGTAA

bioRxiv preprint doi: https://doi.org/10.1101/2025.10.23.684235; this version posted December 4, 2025. The copyright holder for this preprint (which was not certified by peer review) is the author/funder, who has granted bioRxiv a license to display the preprint in perpetuity. It is made available under aCC-BY 4.0 International license.

GGGGGATTTCTGTTCATGGGGGTAATGATACCGATGAAACGAGAGAGGATG
CTCACGATACGGGTTACTGATGATGAACATGCCCGGTTACTGGAACGTTGT
GAGGGTAAACAACTGGCGGTATGGATGCGGCGGGACCAGAGAAAAATCAC
TCAGGGTCAATGCCAGCGCTTCGTTAATACAGATGTAGGTGTTCCACAGGG
TAGCCAGCAGCATCCTGCGATGCAGATCCGGAACATAATGGTGCAGGGCG
CTGACTTCCGCGTTTCCAGACTTTACGAAACACGGAAACCGAAGACCATTCA
TGTTGTTGCTCAGGTCGCAGACGTTTTGCAGCAGCAGTCGCTTCACGTTCG
CTCGCGTATCGGTGATTCATTCTGCTAACCAGTAAGGCAACCCCGCCAGCC
TAGCCGGGTCCTCAACGACAGGAGCACGATCATGCGCACCCGTGGCCAGG
ACCCAACGCTGCCCGAGATGCGCCGCGTGCGGCTGCTGGAGATGGCGGA
CGCGATGGATATGTTCTGCCAAGGGTTGGTTTGCGCATTCACAGTTCTCCG
CAAGAATTGATTGGCTCCAATTCTTGGAGTGGTGAATCCGTTAGCGAGGTG
CCGCCGGCTTCCATTCAGGTCGAGGTGGCCCGGCTCCATGCACCGCGACG
CAACGCGGGGAGGCAGACAAGGTATAGGGCGGCGCCTACAATCCATGCCA
ACCCGTTCCATGTGCTCGCCGAGGCGGCATAAATCGCCGTGACGATCAGC
GGTCCAGTGATCGAAGTTAGGCTGGTAAGAGCCGCGAGCGATCCTTGAAG
CTGTCCCTGATGGTCGTCATCTACCTGCCTGGACAGCATGGCCTGCAACGC
GGGCATCCCGATGCCGCCGGAAGCGAGAAGAATCATAATGGGGAAGGCCA
TCCAGCCTCGCGTCGCGAACGCCAGCAAGACGTAGCCCAGCGCGTCGGCC
GCCATGCCGGCGATAATGGCCTGCTTCTCGCCGAAACGTTTGGTGGCGGG
ACCAGTGACGAAGGCTTGAGCGAGGGCGTGCAAGATTCCGAATACCGCAA
GCGACAGGCCGATCATCGTCGCGCTCCAGCGAAAGCGGTCCTCGCCGAAA
ATGACCCAGAGCGCTGCCGGCACCTGTCCTACGAGTTGCATGATAAAGAAG
ACAGTCATAAGTGCGGCGACGATAGTCATGCCCCGCGCCCACCGGAAGGA
GCTGACTGGGTTGAAGGCTCTCAAGGGCATCGGTCGAGATCCCGGTGCCT
AATGAGTGAGCTAACTTACATTAATTGCGTTGCGCTCACTGCCCGCTTTCCA
GTCGGGAAACCTGTCGTGCCAGCTGCATTAATGAATCGGCCAACGCGCGG
GGAGAGGCGGTTTGCGTATTGGGCGCCAGGGTGGTTTTTCTTTTCACCAGT
GAGACGGGCAACAGCTGATTGCCCTTCACCGCCTGGCCCTGAGAGAGTTG
CAGCAAGCGGTCCACGCTGGTTTGCCCCAGCAGGCGAAAATCCTGTTTGAT
GGTGGTTAACGGCGGGATATAACATGAGCTGTCTTCGGTATCGTCGTATCC

bioRxiv preprint doi: https://doi.org/10.1101/2025.10.23.684235; this version posted December 4, 2025. The copyright holder for this preprint (which was not certified by peer review) is the author/funder, who has granted bioRxiv a license to display the preprint in perpetuity. It is made available under aCC-BY 4.0 International license.

CACTACCGAGATATCCGCACCAACGCGCAGCCCGGACTCGGTAATGGCGCGCATTGCGCCCAGCGCCATCTGATCGTTGGCAACCAGCATCGCAGTGGGAACGATGCCCTCATTCAGCATTTGCATGGTTTGTTGAAAACCGGACATGGCACTCCAGTCGCCTTCCCGTTCCGCTATCGGCTGAATTTGATTGCGAGTGAGATATTTATGCCAGCCAGCCAGACGCAGACGCGCCGAGACAGAACTTAATGGGCCCGCTAACAGCGCGATTTGCTGGTGACCCAATGCGACCAGATGCTCCACGCCCAGTCGCGTACCGTCTTCATGGGAGAAAATAATACTGTTGATGGGTGTCTGGTCAGAGACATCAAGAAATAACGCCGGAACATTAGTGCAGGCAGCTTCCACAGCAATGGCATCCTGGTCATCCAGCGGATAGTTAATGATCAGCCCACTGACGCGTTGCGCGAGAAGATTGTGCACCGCCGCTTTACAGGCTTCGACGCCGCTTCGTTCTACCATCGACACCACCACGCTGGCACCCAGTTGATCGGCGCGAGATTTAATCGCCGCGACAATTTGCGACGGCGCGTGCAGGGCCAGACTGGAGGTGGCAACGCCAATCAGCAACGACTGTTTGCCCGCCAGTTGTTGTGCCACGCGGTTGGGAATGTAATTCAGCTCCGCCATCGCCGCTTCCACTTTTTCCCGCGTTTTCGCAGAAACGTGGCTGGCCTGGTTCACCACGCGGGAAACGGTCTGATAAGAGACACCGGCATACTCTGCGACATCGTATAACGTTACTGGTTTCACATTCACCACCCTGAATTGACTCTCTTCCGGGCGCTATCATGCCATACCGCGAAAGGTTTTGCGCCATTCGATGGTGTCCGGGATCTCGACGCTCTCCCTTATGCGACTCCTGCATTAGGAAGCAGCCCAGTAGTAGGTTGAGGCCGTTGAGCACCGCCGCCGCAAGGAATGGTGCATGCAAGGAGATGGCGCCCAACAGTCCCCCGGCCACGGGGCCTGCCACCATACCCACGCCGAAACAAGCGCTCATGAGCCCGAAGTGGCGAGCCCGATCTTCCCCATCGGTGATGTCGGCGATATAGGCGCCAGCAACCGCACCTGTGGCGCCGGTGATGCCGGCCACGATGCGTCCGGCGTAGAGGATCGAGATCTCGATCCCGCGAAATTAATACGACTCACTATAGGGGAATTGTGAGCGGATAACAATTCCCCTCTAGAAATAATTTTGTTTAACTTTAAGAAGGAGATATACCATGGGCAGCAGCCATCATCATCATCATCACAGCAGCGGCGATGATGATGATAAAATGGCGACCTGGGACGAAAAGGCGGTGACCCGTCGTGCGAAAGTTGCGCCGGCGGAGCGTATGAGCAAGTTCCTGCGTCACTTTACCGTGGTTGGTGACGATTACCACGCGTGGAACATCAACTATAAGAAATGGGAGAACGAGGAAGAGGAAGAGGAAGAGGAACAGCCGCCGCCGACCCCGGTTAGCGGCGAGGAAGGCCGTGCGGCGGCGCCGGATGTGGCGCCGGC

bioRxiv preprint doi: https://doi.org/10.1101/2025.10.23.684235; this version posted December 4, 2025. The copyright holder for this preprint (which was not certified by peer review) is the author/funder, who has granted bioRxiv a license to display the preprint in perpetuity. It is made available under aCC-BY 4.0 International license.

GCCGGGTCCGGCGCCGCGTGCGCCGCTGGATTTCCGTGGCATGCTGCGTA
AACTGTTCAGCAGCCACCGTTTTCAAGTTATCATTATCGCGCTGGTGGTTCT
GGATGCGCTGCTGGTGCTGGCGGAGCTGATCCTGGACCTGAAGATTATCCA
GCCGGATAAAAACAACTACGCGGCGATGGTTTTCCACTATATGAGCATCAC
CATTCTGGTGTTCTTTATGATGGAAATCATCTTCAAGCTGTTCGTTTTCCGTC
TGGAGTTCTTTCACCACAAATTCGAAATCCTGGACGCGGTGGTTGTGGTTGT
GAGCTTTATCCTGGATATTGTGCTGCTGTTCCAGGAGCACCAATTTGAAGCG
CTGGGTCTGCTGATTCTGCTGCGTCTGTGGCGTGTTGCGCGTATTATCAAC
GGCATTATCATTAGCGTGAAGACCCGTAGCGAGCGTCAGCTGCTGCGTCTG
AAGCAGATGAACGTTCAACTGGCGGCGAAAATCCAGCACCTGGAATTTAGC
GCGAGCGAGAAGGAACAAGAGATTGAACGTCTGAACAAACTGCTGCGTCAG
CACGGTCTGCTGGGCGAAGTGAACTAAGGATCCGGCTGCTAACAAAGCCC
GAAAGGAAGCTGAGTTGGCTGCTGCCACCGCTGAGCAATAACTAGCATAAC
CCCCTTGGGGCCTCTAAACGGGTCTTGAGGGGTTTTTTGCTGAAAGGAGGA
ACTATATCCGGATATCCCGCAAGAGGCCCGGCAGTACCGGCATAACCAAGC
CTATGCCTACAGCATCCAGGGTGACGGTGCCGAGGATGACGATGAGCGCA
TTGTTAGATTTCATACACGGTGCCTGACTGCGTTAGCAATTTAACTGTGATAA
ACTACCGCATTAAAGCTTATCGATGATAAGCTGTCAAACATGAGAATTCTTG
AAGACGAAAGGGCCTCGTGATACGCCTATTTTTATAGGTTAATGTCATGATA
ATAATGGTTTCTTAGACGTCAGGTGGCACTTTTCGGGGAAATGTGCGCGGA
ACCCCTATTTGTTTATTTTTCTAAATACATTCAAATATGTATCCGCTCATGAGA
CAATAACCCTGATAAATGCTTCAATAATATTGAAAAAGGAAGAGTATGAGTAT
TCAACATTTCCGTGTCGCCCTTATTCCCTTTTTTGCGGCATTTTGCCTTCCTG
TTTTTGCTCACCCAGAAACGCTGGTGAAAGTAAAAGATGCTGAAGATCAGTT
GGGTGCACGAGTGGGTTACATCGAACTGGATCTCAACAGCGGTAAGATCCT
TGAGAGTTTTCGCCCCGAAGAACGTTTTCCAATGATGAGCACTTTTAAAGTT
CTGCTATGTGGCGCGGTATTATCCCGTGTTGACGCCGGGCAAGAGCAACTC
GGTCGCCGCATACACTATTCTCAGAATGACTTGGTTGAGTACTCACCAGTCA
CAGAAAAGCATCTTACGGATGGCATGACAGTAAGAGAATTATGCAGTGCTG
CCATAACCATGAGTGATAACACTGCGGCCAACTTACTTCTGACAACGATCG
GAGGACCGAAGGAGCTAACCGCTTTTTTGCACAACATGGGGGATCATGTAA

bioRxiv preprint doi: https://doi.org/10.1101/2025.10.23.684235; this version posted December 4, 2025. The copyright holder for this preprint (which was not certified by peer review) is the author/funder, who has granted bioRxiv a license to display the preprint in perpetuity. It is made available under aCC-BY 4.0 International license.

CTCGCCTTGATCGTTGGGAACCGGAGCTGAATGAAGCCATACCAAACGACG
AGCGTGACACCACGATGCCTGCAGCAATGGCAACAACGTTGCGCAAACTAT
TAACTGGCGAACTACTTACTCTAGCTTCCCGGCAACAATTAATAGACTGGAT
GGAGGCGGATAAAGTTGCAGGACCACTTCTGCGCTCGGCCCTTCCGGCTG
GCTGGTTTATTGCTGATAAATCTGGAGCCGGTGAGCGTGGGTCTCGCGGTA
TCATTGCAGCACTGGGGCCAGATGGTAAGCCCTCCCGTATCGTAGTTATCT
ACACGACGGGGAGTCAGGCAACTATGGATGAACGAAATAGACAGATCGCTG
AGATAGGTGCCTCACTGATTAAGCATTGGTAACTGTCAGACCAAGTTTACTC
ATATATACTTTAGATTGATTTAAAACTTCATTTTTAATTTAAAAGGATCTAGGT
GAAGATCCTTTTTGATAATCTCATGACCAAAATCCCTTAACGTGAGTTTTCGT
TCCACTGAGCGTCAGACCCCGTAGAAAAGATCAAAGGATCTTC

>MjA9Acd-RS.pDule2
TTGAGATCGTTTTGGTCTGCGCGTAATCTCTTGCTCTGAAAACGAAAAAACC
GCCTTGCAGGGCGGTTTTTCGAAGGTTCTCTGAGCTACCAACTCTTTGAACC
GAGGTAACTGGCTTGGAGGAGCGCAGTCACCAAAACTTGTCCTTTCAGTTT
AGCCTTAACCGGCGCATGACTTCAAGACTAACTCCTCTAAATCAATTACCAG
TGGCTGCTGCCAGTGGTGCTTTTGCATGTCTTTCCGGGTTGGACTCAAGAC
GATAGTTACCGGATAAGGCGCAGCGGTCGGACTGAACGGGGGGTTCGTGC
ATACAGTCCAGCTTGGAGCGAACTGCCTACCCGGAACTGAGTGTCAGGCGT
GGAATGAGACAAACGCGGCCATAACAGCGGAATGACACCGGTAAACCGAA
AGGCAGGAACAGGAGAGCGCACGAGGGAGCCGCCAGGGGGAAACGCCTG
GTATCTTTATAGTCCTGTCGGGTTTCGCCACCACTGATTTGAGCGTCAGATT
TCGTGATGCTTGTCAGGGGGGCGGAGCCTATGGAAAAACGGCTTTGCCGC
GGCCCTCTCACTTCCCTGTTAAGTATCTTCCTGGCATCTTCCAGGAAATCTC
CGCCCCGTTCGTAAGCCATTTCCGCTCGCCGCAGTCGAACGACCGAGCGT
AGCGAGTCAGTGAGCGAGGAAGCGGAATATATCCTGTATCACATATTCTGC
TGACGCACCGGTGCAGCCTTTTTTCTCCTGCCACATGAAGCACTTCACTGAC
ACCCTCATCAGTGCCAACATAGTAAGCCAGTATACACTCCGCTAGCGCTGA
TGTCCGGCGGTGCTTTTGCCGTTACGCACCACCCCGTCAGTAGCTGAACAG
GAGGGACAGCTCCCGGCGGATTTGTCCTACTCAGGAGAGCGTTCACCGAC

bioRxiv preprint doi: https://doi.org/10.1101/2025.10.23.684235; this version posted December 4, 2025. The copyright holder for this preprint (which was not certified by peer review) is the author/funder, who has granted bioRxiv a license to display the preprint in perpetuity. It is made available under aCC-BY 4.0 International license.

AAACAACAGATAAAACGAAAGGCCCAGTCTTTCGACTGAGCCTTTCGTTTTA
TTTGATGCCTGGCAGTTCCCTACTCTCGCATGGGGAGACCCCACACTACCA
TCGGCGCTACGGCGTTTCACTTCTGAGTTCGGCATGGGGTCAGGTGGGAC
CACCGCGCTACTGCCGCCAGGCAAATTCTGTTTTATCAGACCGCTTCTGCG
TTCTGATTTAATCTGTATCAGGCTGAAAATCTTCTCTCATCCGCCAAAACAGC
CAAGCTGGAGACCGTTTAAACTCAATGATGATGATGATGATGGTCGACGGC
GCTATTCAGATCCTCTTCTGAGATGAGTTTTTGTTCGGGCCCAAGCTTCGAA
TTCCCATATGGTACCCGTTTGAAACTGCAGTTATAATCTCTTTCTAATTGGCT
CTAAAATCTTTATAAGTTCTTCAGCTACAGCATTTTTTAAATCCATTGGATGC
AATTCCTTATTTTTAAATAAACTCTCTAACTCCTCATAGCTATTAACTGTCAAA
TCTCCACCAAATTTTTCTGGCCTTTTTATGGTTAAAGGATATTCAAGGAAGTA
TTTAGCTATCTCCATTATTGGATTTCCTTCAACAACTCCAGCTGGGCAGTATG
CTTTCTTTATCTTAGCCCTAATCTCTTCTGGAGAGTCATCAACAGCTATAAAA
TTCCCTTTTGAAGAACTCATCTTTCCTTCTCCATCCAAACCCGTTAAGACAGG
GTTGTGAATACAAACAACCTTTTTTGGTAAAAGCTCCCTTGCTAACATGTTTA
TTTTTCTCTGCTCCATCCCTCCAACTGCAACATCAACGCCAGTATAATGAATA
GAATTAACCTGCATTATTGGATAGATAACTTCAGCAACCTTTGGATTTTCATC
CTCTCTTGCTATAAGTTCCATACTCCTTCTTGCTCTTTTTAAGGTAGTTTTTAA
AGCCAATCTATAGACATTCAGTGTATAATCCTTATCAAGCTCCAGTTCACTTC
CATAAACATATTTTGCCTTTAACCCCATTGCTTCAAAAACTTTTTTGTTATAAT
CTCCTATTTTTCTAATCTCATCCAACTCTCCTTTCTGGTTTAAATAGGCGTGT
AAATCAGCCAAATCTATAATTATATCAAATCCAGCATTTTGTAAATCAATCAT
CTTTTTTATTTGGAGATAATGCCCTAAATGTATTTTACCACTTGGTTCAAAAC
CTATCGCAGCAGATTTTTCATCTTTTTTTAAAACCTCTCTTAACTCTTCCTCG
CTGATAATTTCAGATGTGTTTCTCTTTATCATTTCAAATTCGTCCATGGGGGA
TTCCTCAAAGCGTAAACTCAGCGTTACAAGTATTACACAAAGTTTTTTATGTT
GAGAATATTTTTTTGATGGGGCGCCACTTATTTTTGATCGTTCGCTCAAAGAA
GCGGCGCCAGGGTTGTTTTTCTTTTCACCGGTGAGACGGGCAACAGAACGC
CATGAGCGGCCTCATTTCTTATTCTGAGTTACAACAGTCCGCACCGCTGTCC
GTATATATGAGTAAACTTGGTCCCGGGTTACCGGTTTGGTTAGCGAGAAGA
GCCAGTAAAAGACGCAGTGACGGCAATGTCTGATGCAATATGGACAATTGG

bioRxiv preprint doi: https://doi.org/10.1101/2025.10.23.684235; this version posted December 4, 2025. The copyright holder for this preprint (which was not certified by peer review) is the author/funder, who has granted bioRxiv a license to display the preprint in perpetuity. It is made available under aCC-BY 4.0 International license.

TTTCTTCTCTGAATGGCGGGAGTATGAAAAGTATGGCTGAAGCGCAAAATGA
TCCCCTGCTGCCGGGATACTCGTTTAATGCCCATCTGGTGGCGGGTTTAAC
GCCGATTGAGGCCAACGGTTATCTCGATTTTTTTATCGACCGACCGCTGGG
AATGAAAGGTTATATTCTCAATCTCACCATTCGCGGTCAGGGGGTGGTGAAA
AATCAGGGACGAGAATTTGTTTGCCGACCGGGTGATATTTTGCTGTTCCCG
CCAGGAGAGATTCATCACTACGGTCGTCATCCGGAGGCTCGCGAATGGTAT
CACCAGTGGGTTTACTTTCGTCCGCGCGCCTACTGGCATGAATGGCTTAAC
TGGCCGTCAATATTTGCCAATACGGGGTTCTTTCGCCCGGATGAAGCGCAC
CAGCCGCATTTCAGCGACCTGTTTGGGCAAATCATTAACGCCGGGCAAGGG
GAAGGGCGCTATTCGGAGCTGCTGGCGATAAATCTGCTTGAGCAATTGTTA
CTGCGGCGCATGGAAGCGATTAACGAGTCGCTCCATCCACCGATGGATAAT
CGGGTACGCGAGGCTTGTCAGTACATCAGCGATCACCTGGCAGACAGCAAT
TTTGATATCGCCAGCGTCGCACAGCATGTTTGCTTGTCGCCGTCGCGTCTG
TCACATCTTTTCCGCCAGCAGTTAGGGATTAGCGTCTTAAGCTGGCGCGAG
GACCAACGTATCAGCCAGGCGAAGCTGCTTTTGAGCACCACCCGGATGCCT
ATCGCCACCGTCGGTCGCAATGTTGGTTTTGACGATCAACTCTATTTCTCGC
GGGTATTTAAAAAATGCACCGGGGCCAGCCCGAGCGAGTTCCGTGCCGGT
TGTGAAGAAAAAGTGAATGATGTAGCCGTCAAGTTGTCATAATTGGTAACGA
ATCAGACAATTGACGGCTTGACGGAGTAGCATAGGGTTTGCAGAATCCCTG
CTTCGTCCATTTGACAGGCACATTATGCATGCCGCTTCGCCTTCGCGCGCG
AATTGATCTGCTGCCTCGCGCGTTTCGGTGATGACGGTGAAAACCTCTGAC
ACATGCAGCTCCCGGAGACGGTCACAGCTTGTCTGTAAGCGGATGCCGGG
AGCAGACAAGCCCGTCAGGGCGCGTCAGCGGGTGTTGGCGGGTGTCGGG
GCGCAGCCATGACCCAGTCAACTGCGATGAGTGGCAGGGCGGGGCGTAAT
TTTTTTAAGGCAGTTATTGGTGCCCTTAAACGCCTGGTTGCTACGCCTGAAT
AAGTGATAATAAGCGGATGAATGGCAGAAATTCGAAAGCAAATTCGACCCG
GTCGTCGGTTCAGGGCAGGGTCGTTAAATAGCCGCTTATGTCTATTGCTGG
TTTACCGGTTTATTGACTACCGGAAGCAGTGTGACCGTGTGCTTCTCAAATG
CCTGAGGCCAGTTTGCTCAGGCTCTCCCCGTGGAGGTAATAATTGACGATA
TGATCATTTATTCTGCCTCCCAGAGCATGATAAAAACGGTTAGCGCTTCGTT
AATACAGATGTAGGTGTTCCACAGGGTAGCCAGCAGCATCCTGCGATGCAG

bioRxiv preprint doi: https://doi.org/10.1101/2025.10.23.684235; this version posted December 4, 2025. The copyright holder for this preprint (which was not certified by peer review) is the author/funder, who has granted bioRxiv a license to display the preprint in perpetuity. It is made available under aCC-BY 4.0 International license.

ATCCGGAACATAATGGTGCAGGGCGCTTGTTTCGGCGTGGGTATGGTGGCA
GGCCCCGTGGCCGGGGGACTGTTGGGCGCTGCCGGCACCTGTCCTACGA
GTTGCATGATAAAGAAGACAGTCATAAGTGCGGCGACGATAGTCATGCCCC
GCGCCCACCGGAAGGAGCTACCGGCAGCGGTGCGGACTGTTGTAACTCAG
AATAAGAAATGAGGCCGCTCATGGCGTTCTGTTGCCCGTCTCACTGGTGAA
AAGAAAAACAACCCTGGCGCCGCTTCTTTGAGCGAACGATCAAAAATAAGT
GGCGCCCCATCAAAAAAATATTCTCAACATAAAAAACTTTGTGTAATACTTGT
AACGCTGAATTCCCGGCGGTAGTTCAGCAGGGCAGAACGGCGGACTCTAA
ATCCGCATGGCGCTGGTTCAAATCCGGCCCGCCGGACCACTGCAGATCCTT
AGCGAAAGCTAAGGATTTTTTTTAAGCTTGGCACTGGCCGTCGTTTTACAAC
GTCGTGACTGGGAAAACCCTGGCGTTACCCAACTTAATCGCCTTGCAGCAC
ATCCCCCTTTCGCCAGACGCTCTCCCTTATGCGACTCCTGCATTAGGAAGC
AGCCCAGTAGTAGGTTGAGGCCGTTGAGCACCGCCGCCGCAAGGAATGGT
GCATGCAAGGAGCCCGAGATGCGCCGCGTGCGGCTGCTGGAGATGGCGG
ACGCGATGGATATGTTCTGCCAAGGGTTGGTTTGCGCATTCACAGTTCTCC
GCAAGAATTGATTGGCTCCAATTCTTGGAGTGGTGAATCCGTTAGCGAGGT
GCCGCCGGCTTCCATTCAGGTCGAGGTGGCCCGGCTCCATGCACCGCGAC
GCAACGCGGGGAGGCAGACAAGGTATAGGGCGGCGCCTACAATCCATGCC
AACCCGTTCCATGTGCTCGCCGAGGCGGCATAAATCGCCGTGACGATCAG
CGGTCCAATGATCGAAGTTAGGCTGGTAAGAGCCGCGAGCGATCCTTGAAG
CTGTCCCTGATGGTCGTCATCTACCTGCCTGGACAGCATGGCCTGCAACGC
GGGCATCCCGATGCCGCCGGAAGCGAGAAGAATCATAATGGGGAAGGCCA
TCCAGCCTCGCGTCGCGAACGCCAGCAAGACGTAGCCCAGCGCGTCGGCC
GCCATGCCGGCGATAATGGCCTGCTTCTCGCCGAAACGTTTGGTGGCGGG
ACCAGTGACGAAGGCTTGAGCGAGGGCGTGCAAGATTCCGAATACCGCAA
GCGACAGGCCGATCATCGTCGCGCTCCAGCGAAAGCGGTCCTCGCCGAAA
ATGACCCAGAGCGCTGCCGGCACCTGTCCTACGAGTTGCATGATAAAGAAG
ACAGTCATAAGTGCGGCGACGATAGTCATGCCCCGCGCCCACCGGAAGGA
GCTGACTGGGTTGAAGGCTCTCAAGGGCATCGGTCGACGCTCTCCCTTATG
CGACTCCTGCATTAGGAAGCAGCCCAGTAGTAGGTTGAGGCCGTTGAGCAC
CGCCGCCGCAAGGAATGGTGCATGCAAGGAGATGGCGCCCAACAGTCCCC

bioRxiv preprint doi: https://doi.org/10.1101/2025.10.23.684235; this version posted December 4, 2025. The copyright holder for this preprint (which was not certified by peer review) is the author/funder, who has granted bioRxiv a license to display the preprint in perpetuity. It is made available under aCC-BY 4.0 International license.

CGGCCACGGGGCCTGCCACCATACCCACGCCGAAACAAGCGCTCATGAGC
CCGAAGTGGCGAGCCCGATCTTCCCCATCGGTGATGTCGGCGATATAGGC
GCCAGCAACCGCACCTGTGGCGCCGGTGATGCCGGCCACGATGCGTCCG
GCGTAGAGGATCCCGGTAAACCAGCAATAGACATAAGCGGCTATTTAACGA
CCCTGCCCTGAACCGACGACCGGGTCATCGTGGCCGGATCTTGCGGCCCC
TCGGCTTGAACGAATTGTTAGACATTATTTGCCGACTACCTTGGTGATCTCG
CCTTTCACGTAGTGGACAAATTCTTCCAACTGATCTGCGCGCGAGGCCAAG
CGATCTTCTTCTTGTCCAAGATAAGCCTGTCTAGCTTCAAGTATGACGGGCT
GATACTGGGCCGGCAGGCGCTCCATTGCCCAGTCGGCAGCGACATCCTTC
GGCGCGATTTTGCCGGTTACTGCGCTGTACCAAATGCGGGACAACGTAAGC
ACTACATTTCGCTCATCGCCAGCCCAGTCGGGCGGCGAGTTCCATAGCGTT
AAGGTTTCATTTAGCGCCTCAAATAGATCCTGTTCAGGAACCGGATCAAAGA
GTTCCTCCGCCGCTGGACCTACCAAGGCAACGCTATGTTCTCTTGCTTTTGT
CAGCAAGATAGCCAGATCAATGTCGATCGTGGCTGGCTCGAAGATACCTGC
AAGAATGTCATTGCGCTGCCATTCTCCAAATTGCAGTTCGCGCTTAGCTGGA
TAACGCCACGGAATGATGTCGTCGTGCACAACAATGGTGACTTCTACAGCG
CGGAGAATCTCGCTCTCTCCAGGGGAAGCCGAAGTTTCCAAAAGGTCGTTG
ATCAAAGCTCGCCGCGTTGTTTCATCAAGCCTTACGGTCACCGTAACCAGC
AAATCAATATCACTGTGTGGCTTCAGGCCGCCATCCACTGCGGAGCCGTAC
AAATGTACGGCCAGCAACGTCGGTTCGAGATGGCGCTCGATGACGCCAACT
ACCTCTGATAGTTGAGTCGATACTTCGGCGATCACCGCTTCCCTCATACTCT
TCCTTTTTCAATATTATTGAAGCATTTATCAGGGTTATTGTCTCATGAGCGGA
TACATATTTGAATGTATTTAGAAAAATAAACAAATAGCTAGCTCACTCGGTCG
CATCGATGATAAGCTGTCAAACATGAGAATTACAACTTATATCGTATGGGGC
TGACTTCAGGTGCTACATTTGAAGAGATAAATTGCACTGAAATCTAGAAATAT
TTTATCTGATTAATAAGATGATCTTC

bioRxiv preprint doi: https://doi.org/10.1101/2025.10.23.684235; this version posted December 4, 2025. The copyright holder for this preprint (which was not certified by peer review) is the author/funder, who has granted bioRxiv a license to display the preprint in perpetuity. It is made available under aCC-BY 4.0 International license.

Supplementary Figures

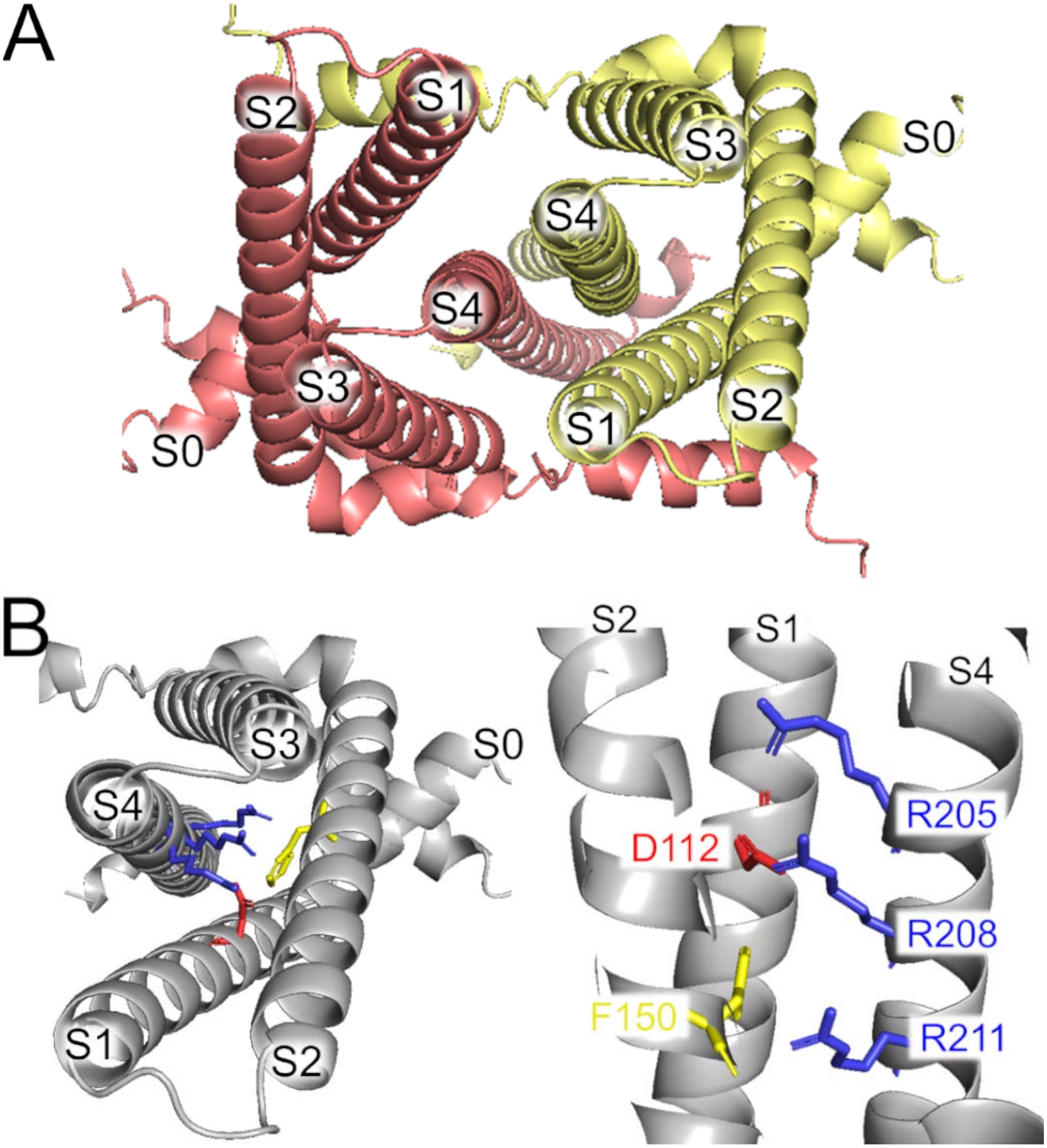

Figure S1. AlphaFold dimer hH$_{v}$1 structural model. (A) Extracellular view of the model, showing each subunit in a different color. (B) One subunit of the dimer showing the position of the arginine gating charges in S4 (blue), the selectivity filter (red), and the charge transfer center (yellow).

bioRxiv preprint doi: https://doi.org/10.1101/2025.10.23.684235; this version posted December 4, 2025. The copyright holder for this preprint (which was not certified by peer review) is the author/funder, who has granted bioRxiv a license to display the preprint in perpetuity. It is made available under aCC-BY 4.0 International license.

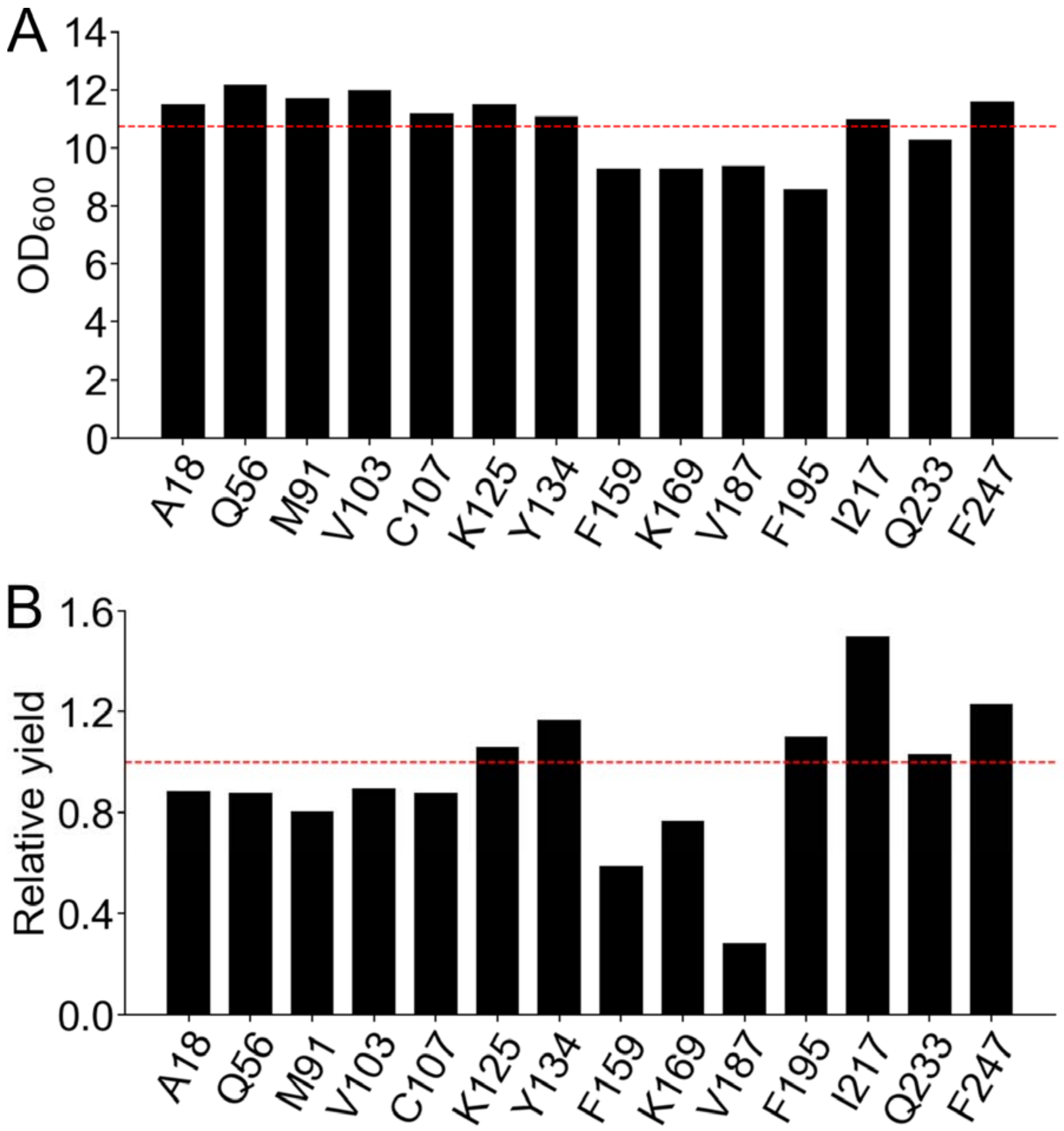

Figure S2. The protein yield of hH_v1-Acd depended on the position of Acd incorporation. (A) Optical density at 600 nm (OD_{600}) of the final cultures expressing the hH_v1 with Acd replacing the indicated amino acid. (B) Relative protein yield calculated by multiplying the OD_{600} and Western blot band intensity of cellular extracts from the final cultures expressing the hH_v1 with Acd replacing the indicated amino acid. Values of normalized by the No TAG condition, which are cells expressing the hH_v1 without any amber stop codon in the presence of Acd and the aminoacyl-tRNA synthetase/tRNA pair (red broken line).

bioRxiv preprint doi: https://doi.org/10.1101/2025.10.23.684235; this version posted December 4, 2025. The copyright holder for this preprint (which was not certified by peer review) is the author/funder, who has granted bioRxiv a license to display the preprint in perpetuity. It is made available under aCC-BY 4.0 International license.

bioRxiv preprint doi: https://doi.org/10.1101/2025.10.23.684235; this version posted December 4, 2025. The copyright holder for this preprint (which was not certified by peer review) is the author/funder, who has granted bioRxiv a license to display the preprint in perpetuity. It is made available under aCC-BY 4.0 International license.

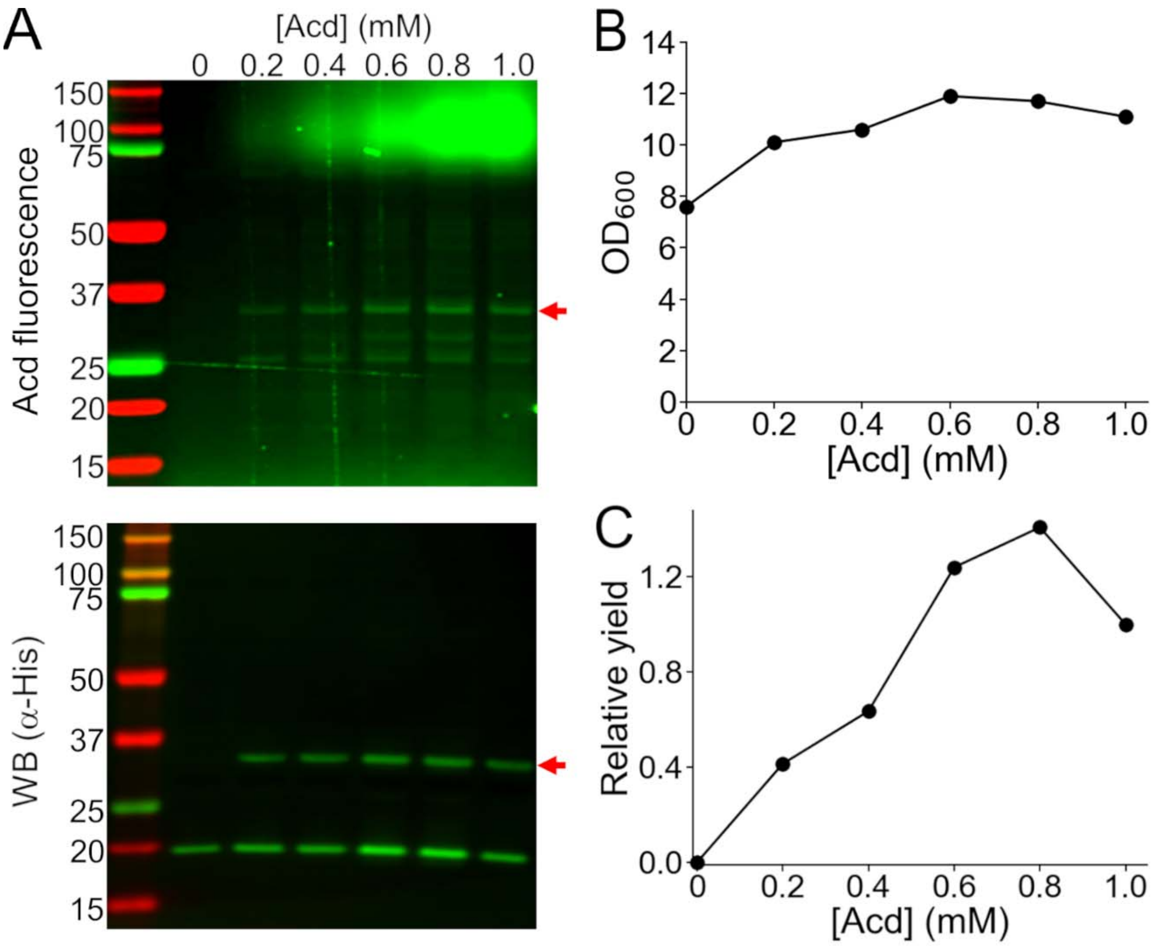

Figure S3. The truncation of hH$_V$1-K125Acd was not decreased at higher concentrations of Acd in the culture medium. (A) Cellular extracts of cells co-transformed with the MjA9-AcdRS/tRNA pair plasmid and hH$_V$1-K125TAG and grown with the indicated concentration of Acd in the culture medium were separated by SDS-PAGE to visualize the hH$_V$1 band (red arrow) by Acd fluorescence (top) and Western blot against the N-terminus His-tag (bottom). (B) Optical density at 600 nm (OD_{600}) of the cultures at the end of the expression. (C) Relative yield obtained by multiplying the Western blot band intensity by the OD600, normalized by the value obtained at 1 mM Acd.

bioRxiv preprint doi: https://doi.org/10.1101/2025.10.23.684235; this version posted December 4, 2025. The copyright holder for this preprint (which was not certified by peer review) is the author/funder, who has granted bioRxiv a license to display the preprint in perpetuity. It is made available under aCC-BY 4.0 International license.

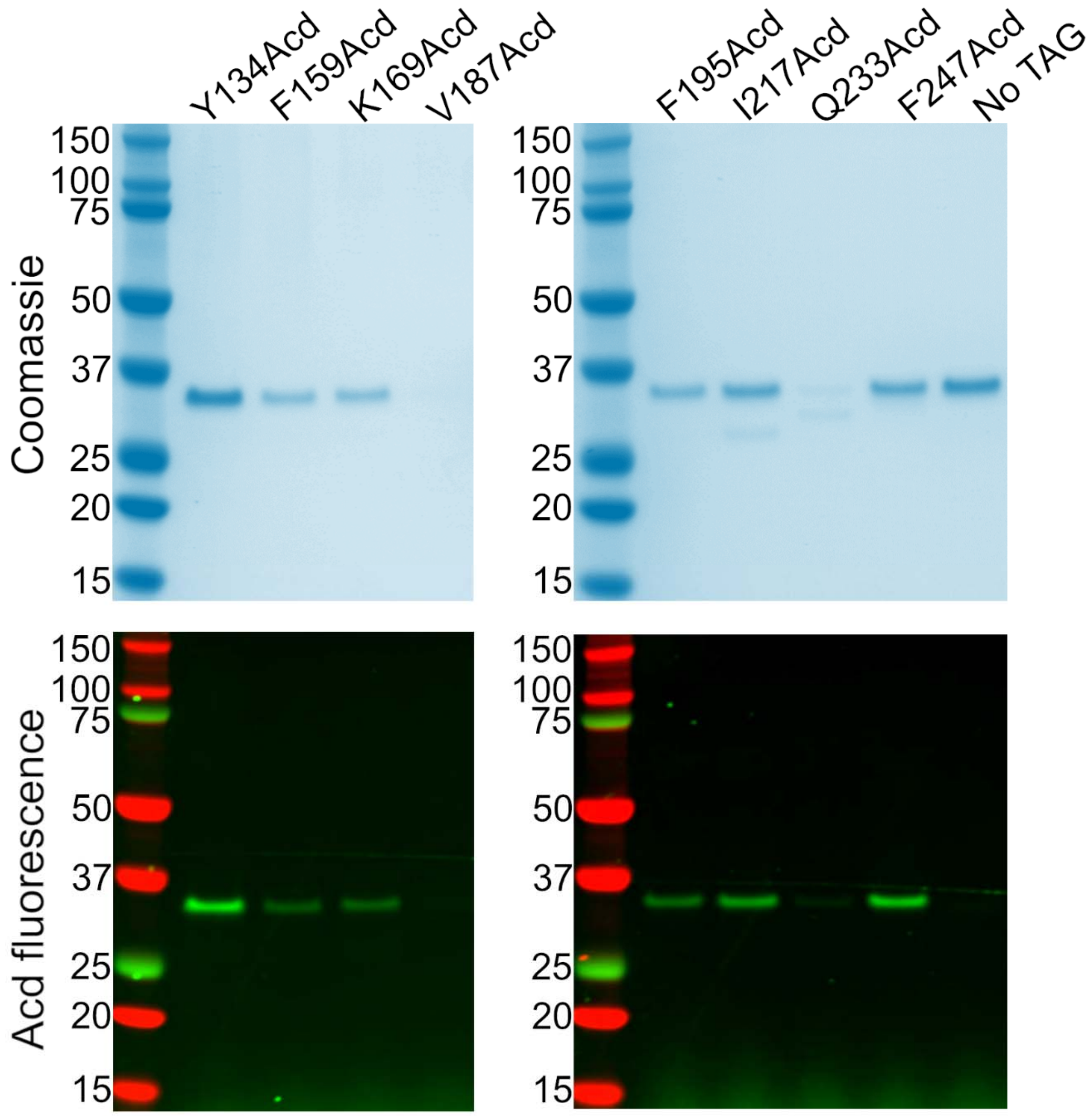

Figure S4. SDS-PAGE of the hH$_v$1 protein samples after purification. Coomassie-stained (top) and Acd fluorescence (bottom) gels of the samples from the elution of the nickel resin column, separated by SDS-PAGE. Note that V187Acd and Q233Acd did not contain appreciable amounts of protein. The No TAG condition consisted of samples purified from cells expressing the hH$_v$1 without any amber stop codon in the presence of Acd and the aminoacyl-tRNA synthetase/tRNA pair.

bioRxiv preprint doi: https://doi.org/10.1101/2025.10.23.684235; this version posted December 4, 2025. The copyright holder for this preprint (which was not certified by peer review) is the author/funder, who has granted bioRxiv a license to display the preprint in perpetuity. It is made available under aCC-BY 4.0 International license.

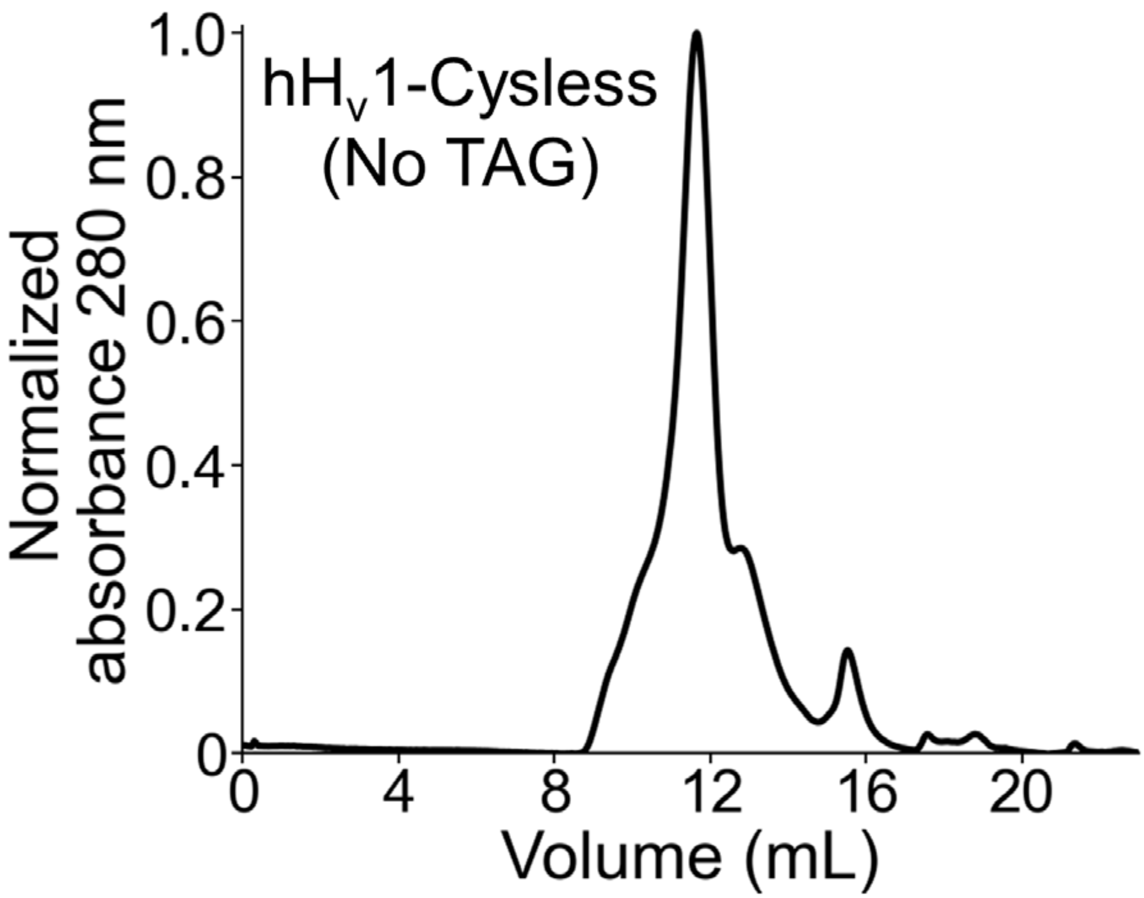

Figure S5. Size exclusion chromatogram of the No TAG sample. The No TAG sample was purified from cells expressing the hH_v1 without any amber stop codon in the presence of Acd and the aminoacyl-tRNA synthetase/tRNA pair. The sample eluted from the immobilized affinity chromatography column was concentrated and injected into the size exclusion chromatography column.

bioRxiv preprint doi: https://doi.org/10.1101/2025.10.23.684235; this version posted December 4, 2025. The copyright holder for this preprint (which was not certified by peer review) is the author/funder, who has granted bioRxiv a license to display the preprint in perpetuity. It is made available under aCC-BY 4.0 International license.

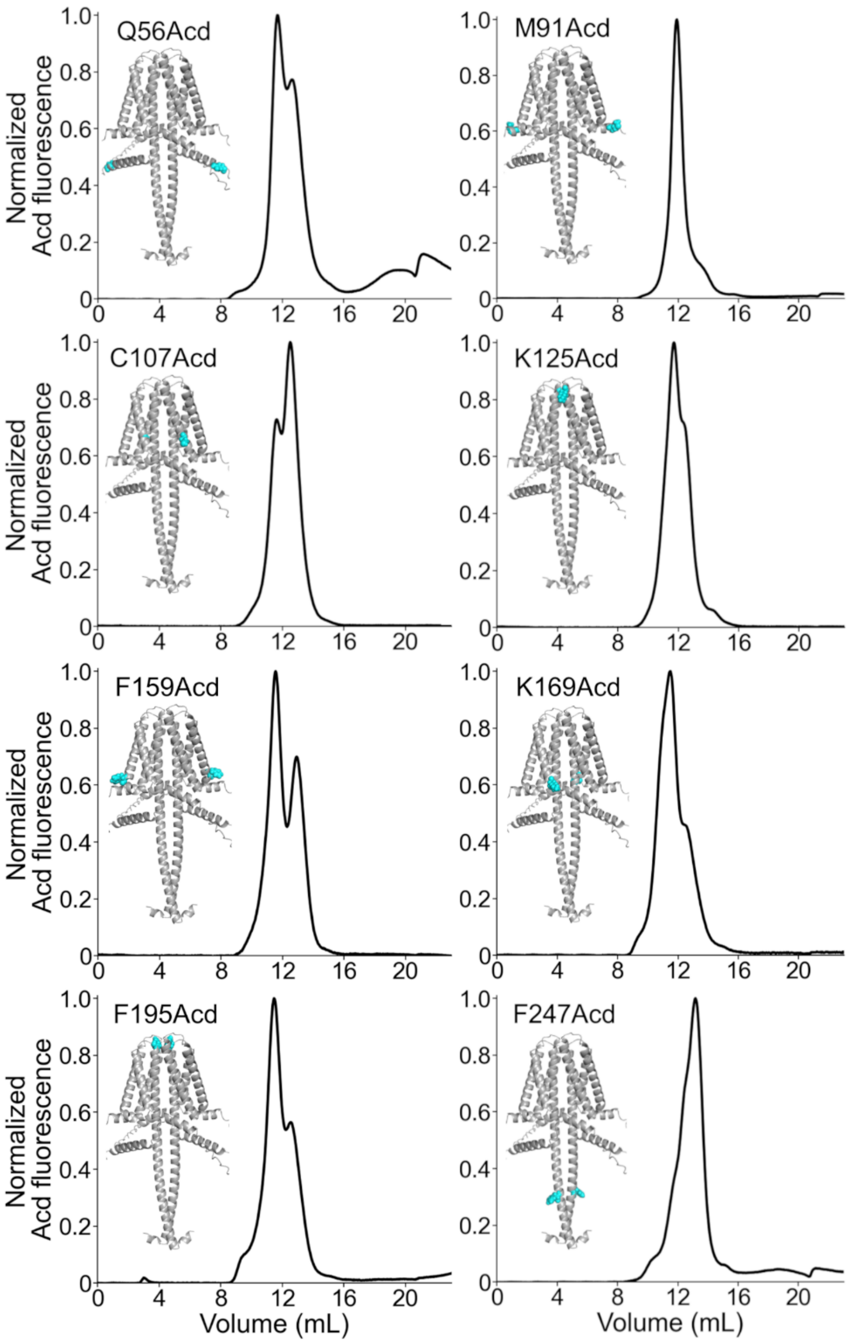

Figure S6. Fluorescence-detection size exclusion chromatography of hH$_v$1-Acd proteins. Chromatograms of the purified hH$_v$1 proteins with Acd incorporated at the indicated amino acid position. The AlphaFold dimer model with the amino acid replaced by Acd as cyan spheres is included.

bioRxiv preprint doi: https://doi.org/10.1101/2025.10.23.684235; this version posted December 4, 2025. The copyright holder for this preprint (which was not certified by peer review) is the author/funder, who has granted bioRxiv a license to display the preprint in perpetuity. It is made available under aCC-BY 4.0 International license.

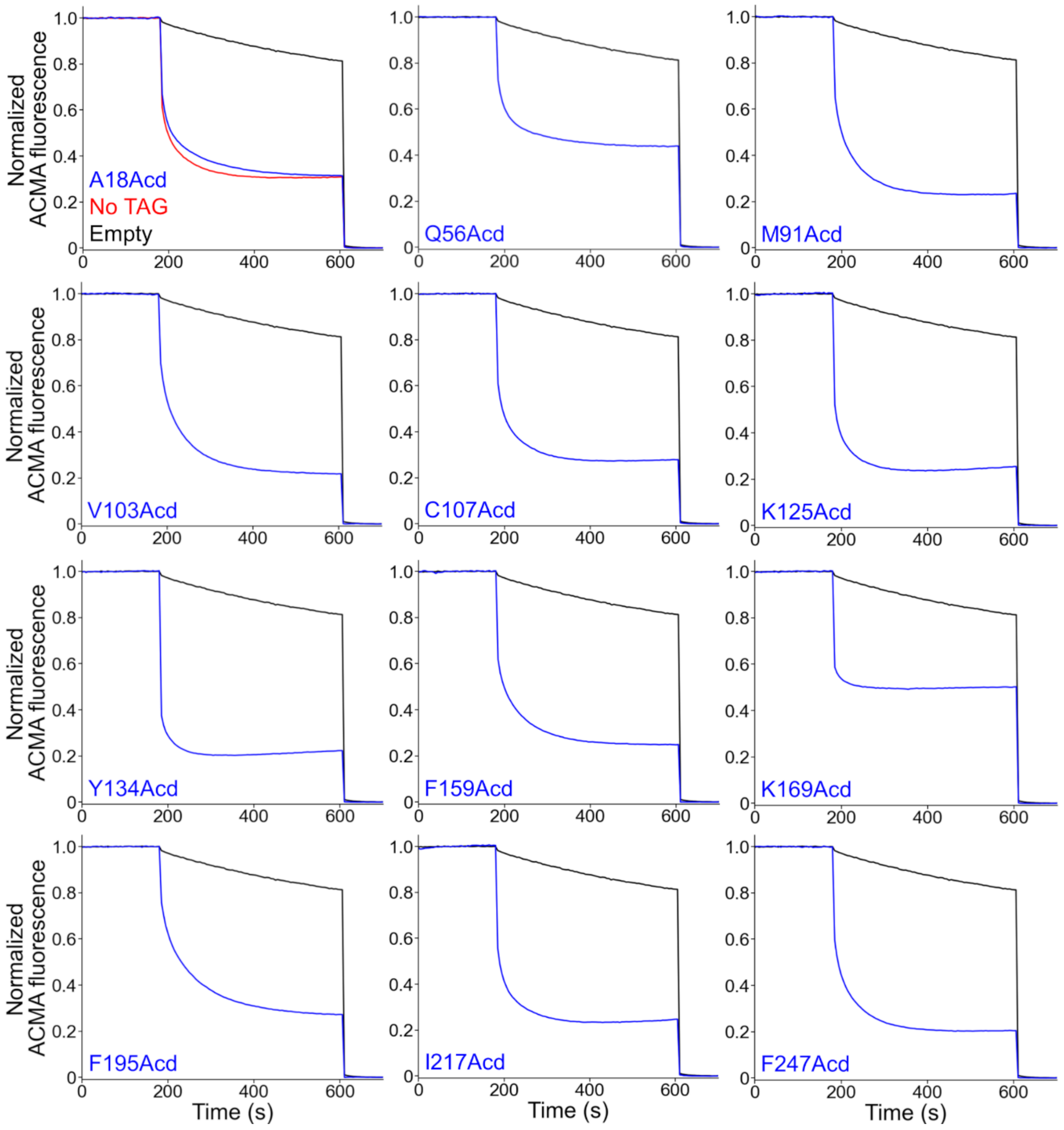

Figure S7. The purified hH$_v$1-Acd proteins were functional proton channels. Liposome proton flux assays of asolectin proteoliposomes containing the indicated hH$_v$1-Acd protein. The proteoliposome samples produced ACMA fluorescence quenching after the addition of valinomycin (180 s) in the presence of a potassium gradient. The protonophore CCCP was added at the end of the experiment (600 s). The No TAG sample (red trace) contains proteoliposomes containing hH$_v$1 without any amber stop codon expressed in the presence of Acd

bioRxiv preprint doi: https://doi.org/10.1101/2025.10.23.684235; this version posted December 4, 2025. The copyright holder for this preprint (which was not certified by peer review) is the author/funder, who has granted bioRxiv a license to display the preprint in perpetuity. It is made available under aCC-BY 4.0 International license.

and the aminoacyl-tRNA synthetase/tRNA pair. Empty asolectin liposomes (black traces) showed slow ACMA fluorescence quenching.

bioRxiv preprint doi: https://doi.org/10.1101/2025.10.23.684235; this version posted December 4, 2025. The copyright holder for this preprint (which was not certified by peer review) is the author/funder, who has granted bioRxiv a license to display the preprint in perpetuity. It is made available under aCC-BY 4.0 International license.

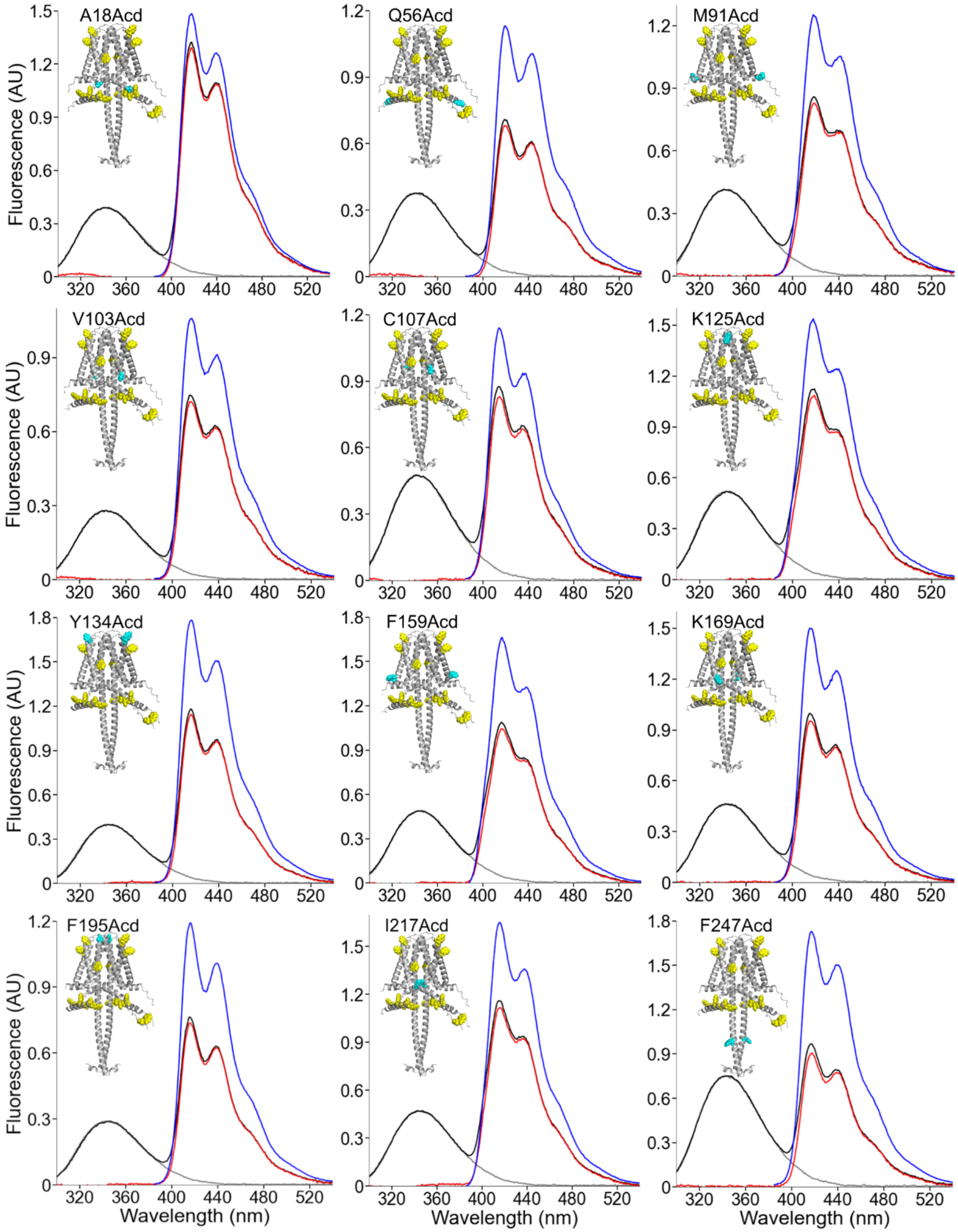

995

Fig. S8. Fluorescence emission spectra of hH_V1-Acd. Spectra of the indicated hH_V1-Acd protein sample obtained when exciting at 280 nm (black) and 370 nm (blue). The isolated spectrum of Acd (red) was obtained by subtracting the black

bioRxiv preprint doi: https://doi.org/10.1101/2025.10.23.684235; this version posted December 4, 2025. The copyright holder for this preprint (which was not certified by peer review) is the author/funder, who has granted bioRxiv a license to display the preprint in perpetuity. It is made available under aCC-BY 4.0 International license.

trace from the normalized spectrum of the No TAG sample excited at 280 nm (grey).

bioRxiv preprint doi: https://doi.org/10.1101/2025.10.23.684235; this version posted December 4, 2025. The copyright holder for this preprint (which was not certified by peer review) is the author/funder, who has granted bioRxiv a license to display the preprint in perpetuity. It is made available under aCC-BY 4.0 International license.

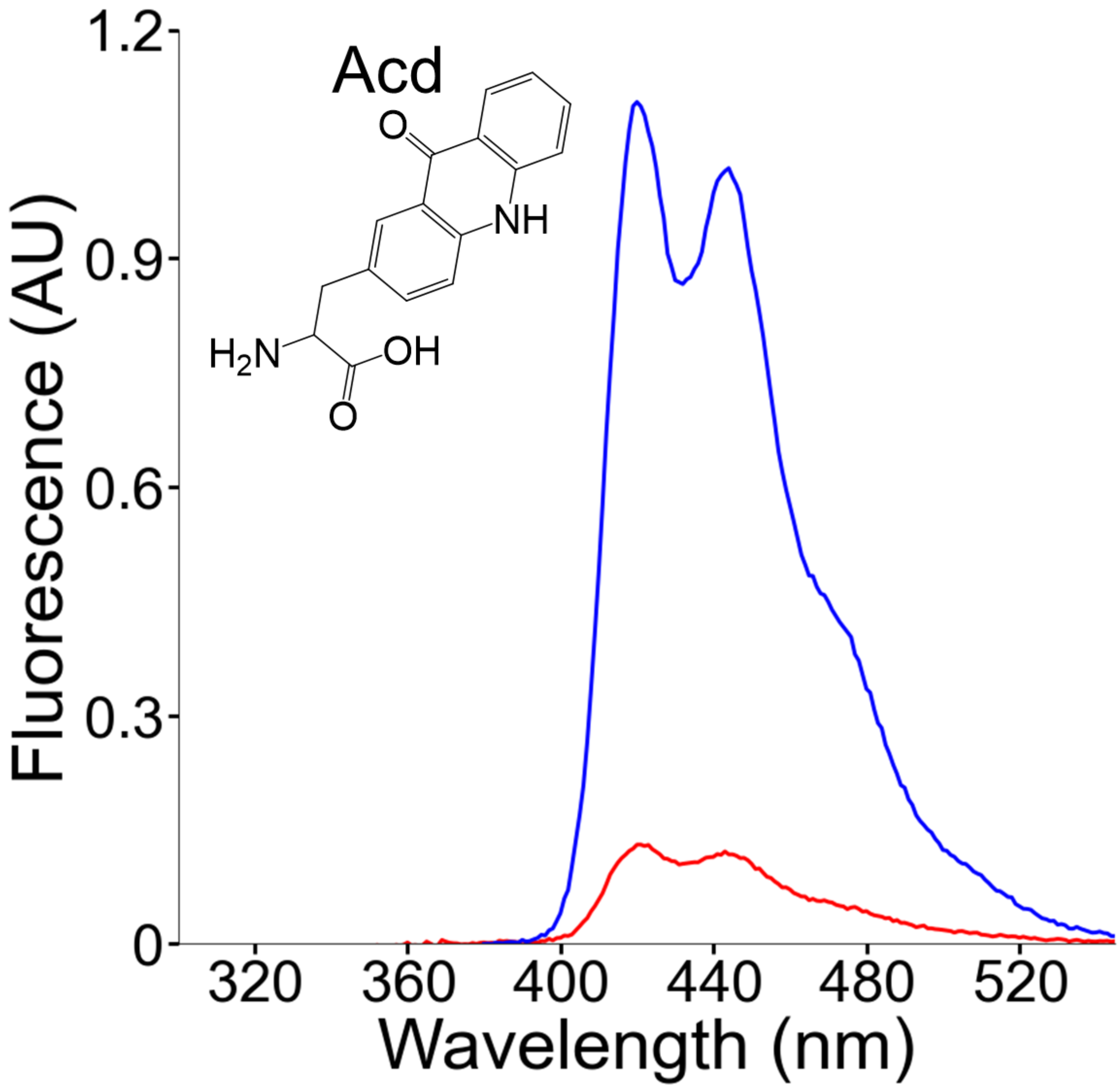

Figure S9. Fluorescence emission spectra of the Acd amino acid in solution. Spectra of Acd in Buffer-H2 using an excitation wavelength of 370 nm (blue) or 280 nm (red).

bioRxiv preprint doi: https://doi.org/10.1101/2025.10.23.684235; this version posted December 4, 2025. The copyright holder for this preprint (which was not certified by peer review) is the author/funder, who has granted bioRxiv a license to display the preprint in perpetuity. It is made available under aCC-BY 4.0 International license.

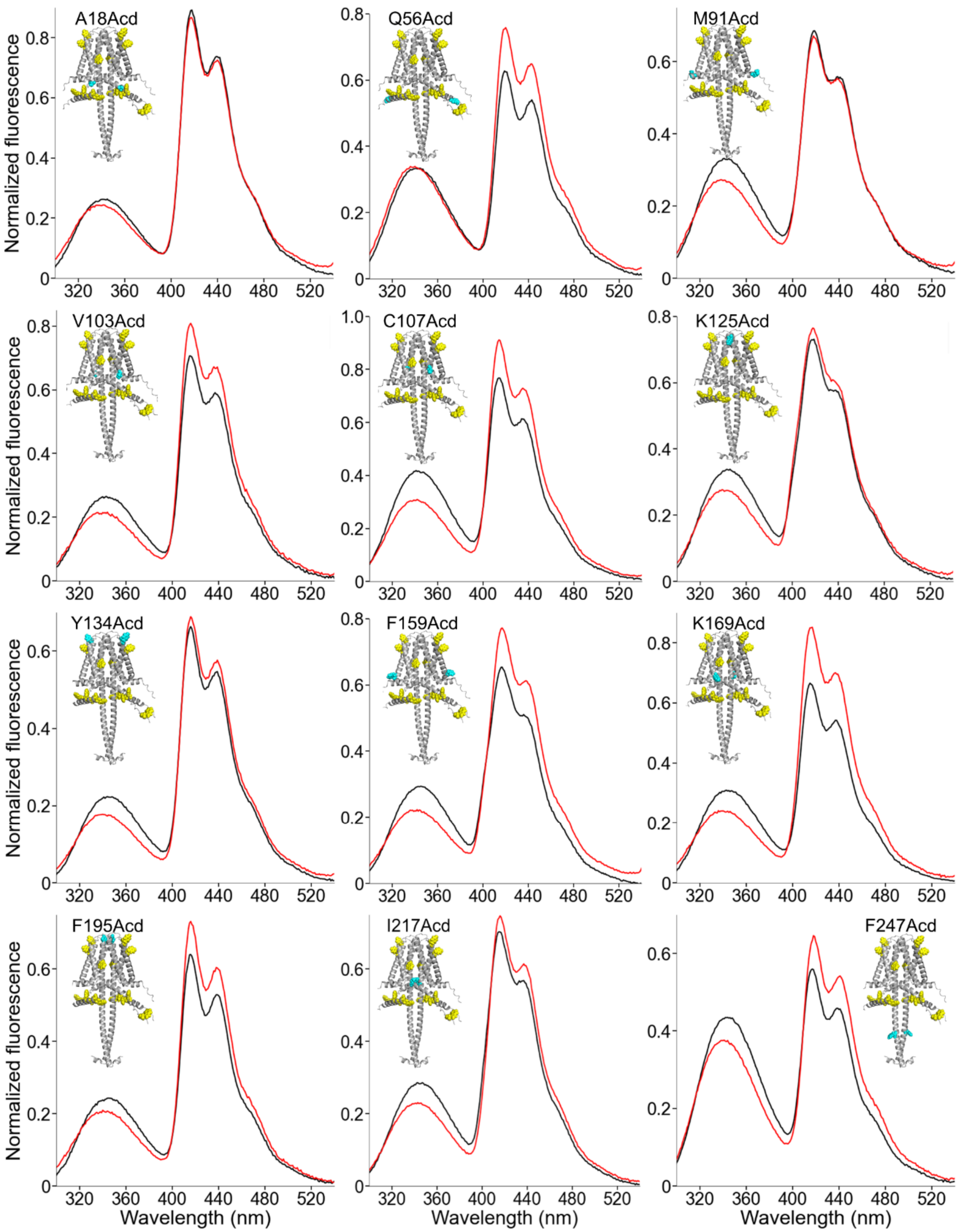

1007

1008 **Fig. S10. Changes in the fluorescence emission spectra of hH$_v$1-Acd in the**
1009 **presence of zinc.** Spectra of the indicated hH$_v$1-Acd protein sample obtained
1010 when exciting at 280 nm in the Apo state (black) and in the presence of 1 mM

bioRxiv preprint doi: https://doi.org/10.1101/2025.10.23.684235; this version posted December 4, 2025. The copyright holder for this preprint (which was not certified by peer review) is the author/funder, who has granted bioRxiv a license to display the preprint in perpetuity. It is made available under aCC-BY 4.0 International license.

Zn^{2+} (red). Spectra were normalized by the maximum intensity of the Acd emission spectrum of the same sample excited at 370 nm.

bioRxiv preprint doi: https://doi.org/10.1101/2025.10.23.684235; this version posted December 4, 2025. The copyright holder for this preprint (which was not certified by peer review) is the author/funder, who has granted bioRxiv a license to display the preprint in perpetuity. It is made available under aCC-BY 4.0 International license.

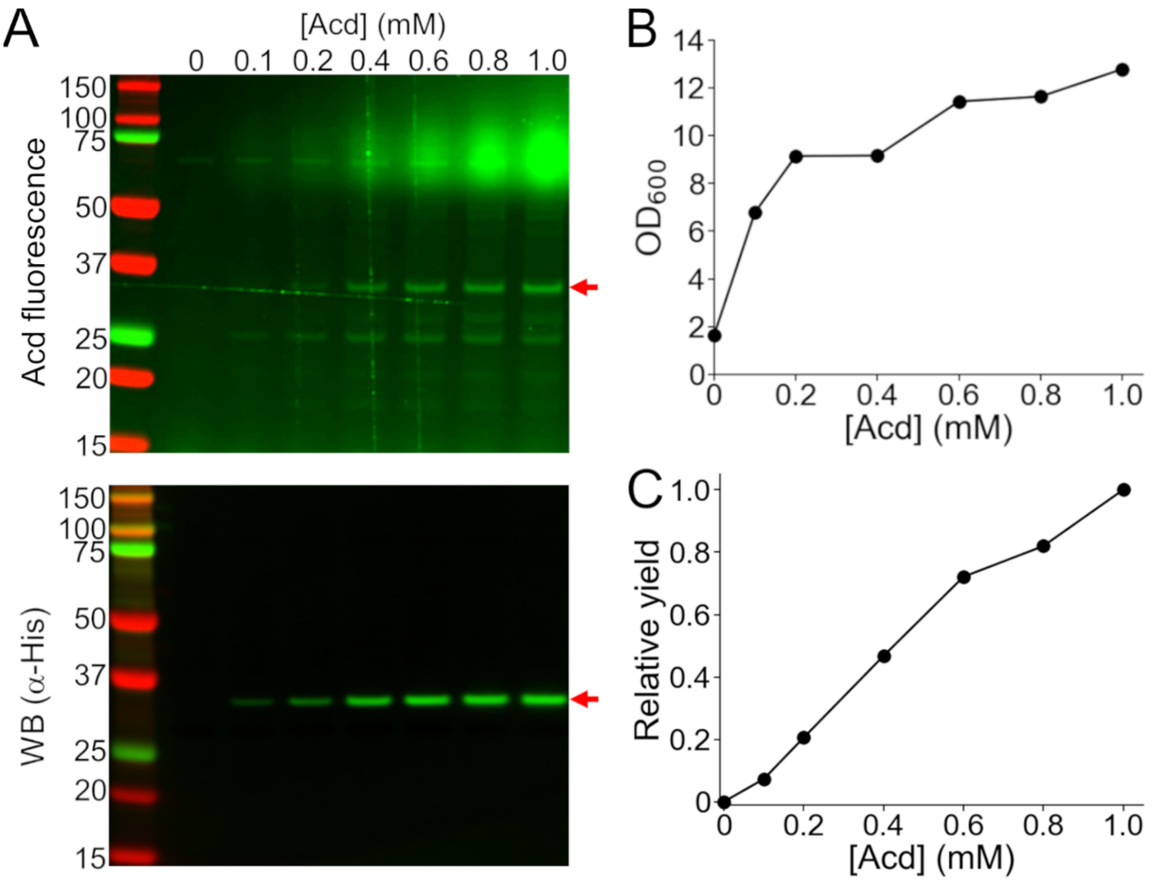

Figure S11. The protein yield of hH$_v$1-A18Acd was proportional to the concentration of Acd in the culture medium. (A) Cellular extracts of cells co-transformed with the MjA9-AcdRS/tRNA pair plasmid and hH$_v$1-A18TAG and grown with the indicated concentration of Acd in the culture medium were separated by SDS-PAGE to visualize the hH$_v$1 band (red arrow) by Acd fluorescence (top) and Western blot against the N-terminus His-tag (bottom). (B) Optical density at 600 nm (OD_{600}) of the cultures at the end of the expression. (C) Relative yield obtained by multiplying the Western blot band intensity by the OD_{600}, normalized by the value obtained at 1 mM Acd.

bioRxiv preprint doi: https://doi.org/10.1101/2025.10.23.684235; this version posted December 4, 2025. The copyright holder for this preprint (which was not certified by peer review) is the author/funder, who has granted bioRxiv a license to display the preprint in perpetuity. It is made available under aCC-BY 4.0 International license.

Supplementary Tables

Table S1. Size-exclusion chromatography elution volumes of the hH$_{V}$1 proteins. Elution volumes were measured as the location of the maximum value of absorbance at 280 nm in the chromatograms.

**hH$_{V}$1 sample**	**Elution volume (mL)**
No TAG	11.5
A18Acd	11.6
Q56Acd	11.7
M91Acd	11.9
V103Acd	11.5
C107Acd	12.5
K125Acd	11.7
Y134Acd	11.5
F159Acd	11.5
K169Acd	11.5
F195 Acd	11.5
I217Acd	12.4
F247Acd	13.1

bioRxiv preprint doi: https://doi.org/10.1101/2025.10.23.684235; this version posted December 4, 2025. The copyright holder for this preprint (which was not certified by peer review) is the author/funder, who has granted bioRxiv a license to display the preprint in perpetuity. It is made available under aCC-BY 4.0 International license.

Table S2. Emission fluorescence spectrum maximum of Acd in different solvents. The wavelength corresponds to the location of the maximum intensity of the emission fluorescence spectrum of the free amino acid dissolved in the corresponding solvent. EtAc: ethyl acetate, OctOH: 1-octanol, ButOH: 1-butanol, EtOH: ethanol.

**Solvent**	**Emission Maximum (nm)**
EtAc	402
OctOH	414
ButOH	415
EtOH	414
Water	421
Buffer-H2	420

bioRxiv preprint doi: https://doi.org/10.1101/2025.10.23.684235; this version posted December 4, 2025. The copyright holder for this preprint (which was not certified by peer review) is the author/funder, who has granted bioRxiv a license to display the preprint in perpetuity. It is made available under aCC-BY 4.0 International license.

Table S3. The emission fluorescence spectrum maximum of the hH_v1-Acd proteins. The wavelength corresponds to the location of the maximum intensity of the emission fluorescence spectrum of the samples.

hH_v1 sample	Emission Maximum (nm)
A18Acd	418
Q56Acd	420
M91Acd	419
V103Acd	417
C107Acd	415
K125Acd	418
Y134Acd	416
F159Acd	418
K169Acd	415
F195 Acd	416
I217Acd	415
F247Acd	418

bioRxiv preprint doi: https://doi.org/10.1101/2025.10.23.684235; this version posted December 4, 2025. The copyright holder for this preprint (which was not certified by peer review) is the author/funder, who has granted bioRxiv a license to display the preprint in perpetuity. It is made available under aCC-BY 4.0 International license.

Supplementary Dataset S1 (separate file). Coordinates of the AlphaFold predicted model of the hH_v1 dimer (in PDB format).

Supplementary References

1. J. R. Lakowicz, Ed., *Principles of Fluorescence Spectroscopy* (Springer US, 2006).

2. M. H. Tessmer, S. Stoll, chiLife: An open-source Python package for in silico spin labeling and integrative protein modeling. *PLoS Comput Biol* **19**, e1010834 (2023).
