# Supplementary material for: Interrogating the structure and function of the human voltage-gated proton channel (hHv1) with a fluorescence noncanonical amino acid
Source: bioRxiv. 2025 Oct 24:2025.10.23.684235. Preprint. [Version 1] doi: 10.1101/2025.10.23.684235 (PMC12633253; doi:10.1101/2025.10.23.684235)
Supplement: Supplementary file 1 [file media-1.pdf]

Supplementary Text

Calculation of the FRET efficiency from the structural model. We calculated the FRET efficiency using the AlphaFold model of the full-length hH$_V$1 (Dataset S1). For each Acd position, the measured steady-state FRET would correspond to the mean FRET efficiency (E_{FRET}), which will be proportional to the *Ratio A – Ratio A$_0$* value obtained from the spectral FRET analysis.

For *n* number of donors and two acceptors in the hH$_V$1 dimer:

$$E_{FRET} = \sum_{i=1}^{n} P_{Ex,i} E_i \quad (1)$$

where $P_{Ex,i}$ is the probability of excitation and E_i is the FRET efficiency of the *i*th donor. $P_{Ex,i}$ is the number of photons absorbed by the *i*th donor divided by the total number of photons absorbed by the total number *n* of donors, which is quantified by the donor's extinction coefficient. Supposing the same extinction coefficient for each of the Trp (ε_W = 5,501 M^{-1} cm^{-1}) and Tyr (ε_Y = 1,209 M^{-1} cm^{-1})(1), equation 1 can be written as:

$$E_{FRET} = \sum_{i=1}^{n} \left(\frac{\varepsilon_i}{\sum_{i=1}^{n} \varepsilon_i}\right) E_i = \frac{1}{n_W \varepsilon_W + n_Y \varepsilon_Y} \left(\varepsilon_W \sum_{i=1}^{n_W} E_{W,i} + \varepsilon_Y \sum_{i=1}^{n_Y} E_{Y,i}\right) \quad (2)$$

where the sum of the total number of Trp (n_W) and Tyr (n_Y) is equal to *n*. The efficiency of FRET for the *i*th donor can be expressed as the sum of rates of energy transfer to each of the *j* = 2 Acd acceptors, k_{ij}, divided by the total rates of photon emission. Therefore, the right terms of equation 2 can be expressed as:

$$\sum_{i=1}^{n_W} E_{W,i} = \sum_{i=1}^{n_W} \left(\frac{\sum_{j=1}^{2} k_{ij}}{\sum_{j=1}^{2} k_{ij} + (1/\tau_W)}\right) \text{ and } \sum_{i=1}^{n_Y} E_{Y,i} = \sum_{i=1}^{n_Y} \left(\frac{\sum_{j=1}^{2} k_{ij}}{\sum_{j=1}^{2} k_{ij} + (1/\tau_Y)}\right) \quad (3)$$

where the Trp lifetime (τ_W = 3.1 ns) and tyrosine lifetime (τ_Y = 3.6 ns) in the absence of donors are considered constant(1). Finally, each energy transfer rate k_{ij} is a function of the distance r_{ij} between the *i*th donor and the *j*th acceptor:

$$k_{ij}(r) = \frac{1}{\tau_D} \left(\frac{R_0}{r_{ij}}\right)^6 \quad (4)$$

where the donor lifetime τ_D and R_0 would be τ_W and 23.5 Å or τ_Y and 20.9 Å for Trp or Tyr residues, respectively. Calculating E_{FRET} using the above equations considers a fixed distance between donors and acceptors. A better approach is to consider the distance distributions $P_{ij}(r)$ produced by the different rotameric states of the FRET pair, which can be modeled easily using chiLife(2). Then, equation 4 will be replaced by equation 5:

$$k_{ij}(r) = \int_0^{\infty} P_{ij}(r) \left[\frac{1}{\tau_D} \left(\frac{R_0}{r}\right)^6\right] dr \quad (5).$$

Supplementary Figures

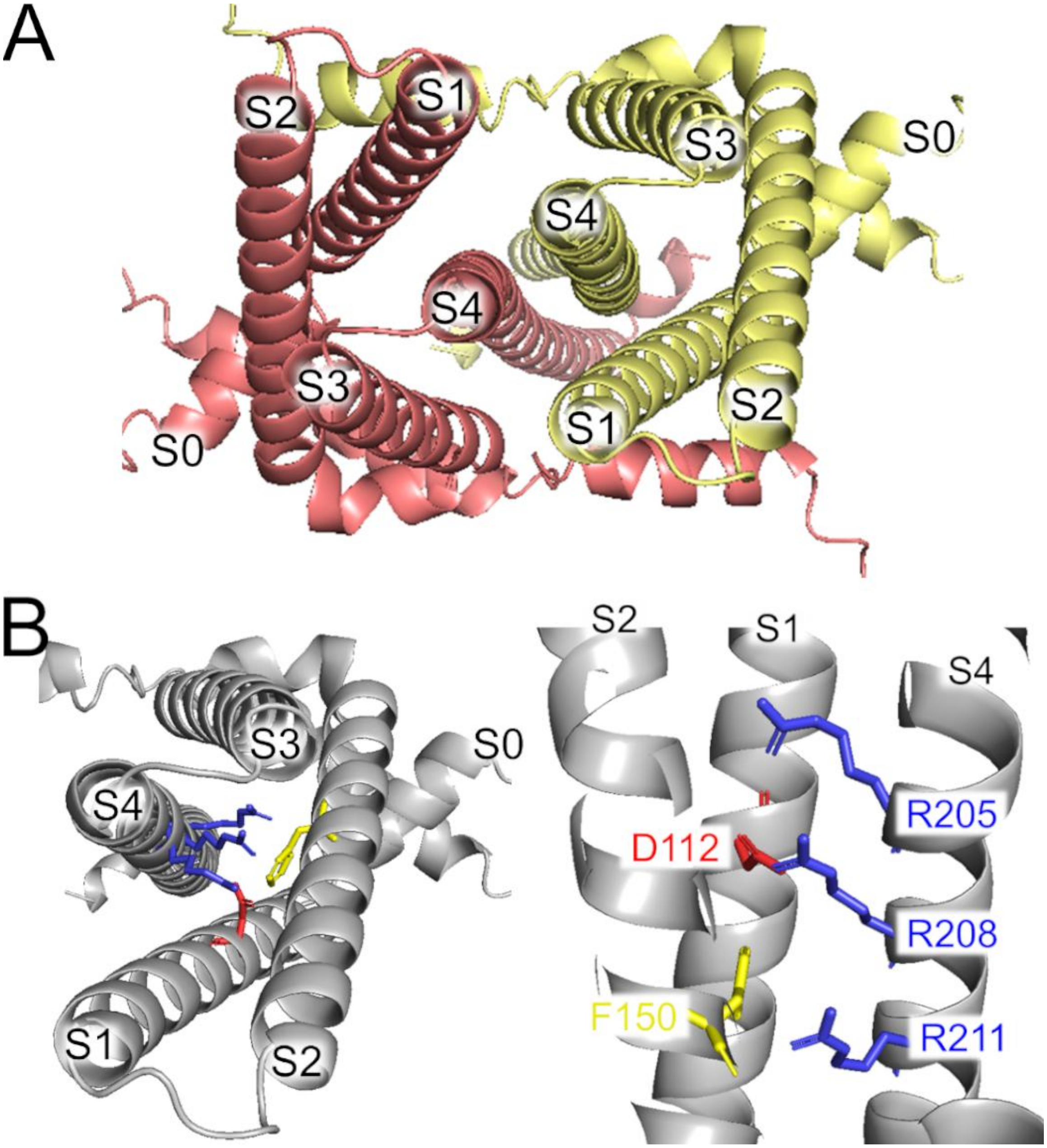

Figure S1. AlphaFold dimer hH$_v$1 structural model. (A) Extracellular view of the model, showing each subunit in a different color. (B) One subunit of the dimer showing the position of the arginine gating charges in S4 (blue), the selectivity filter (red), and the charge transfer center (yellow).

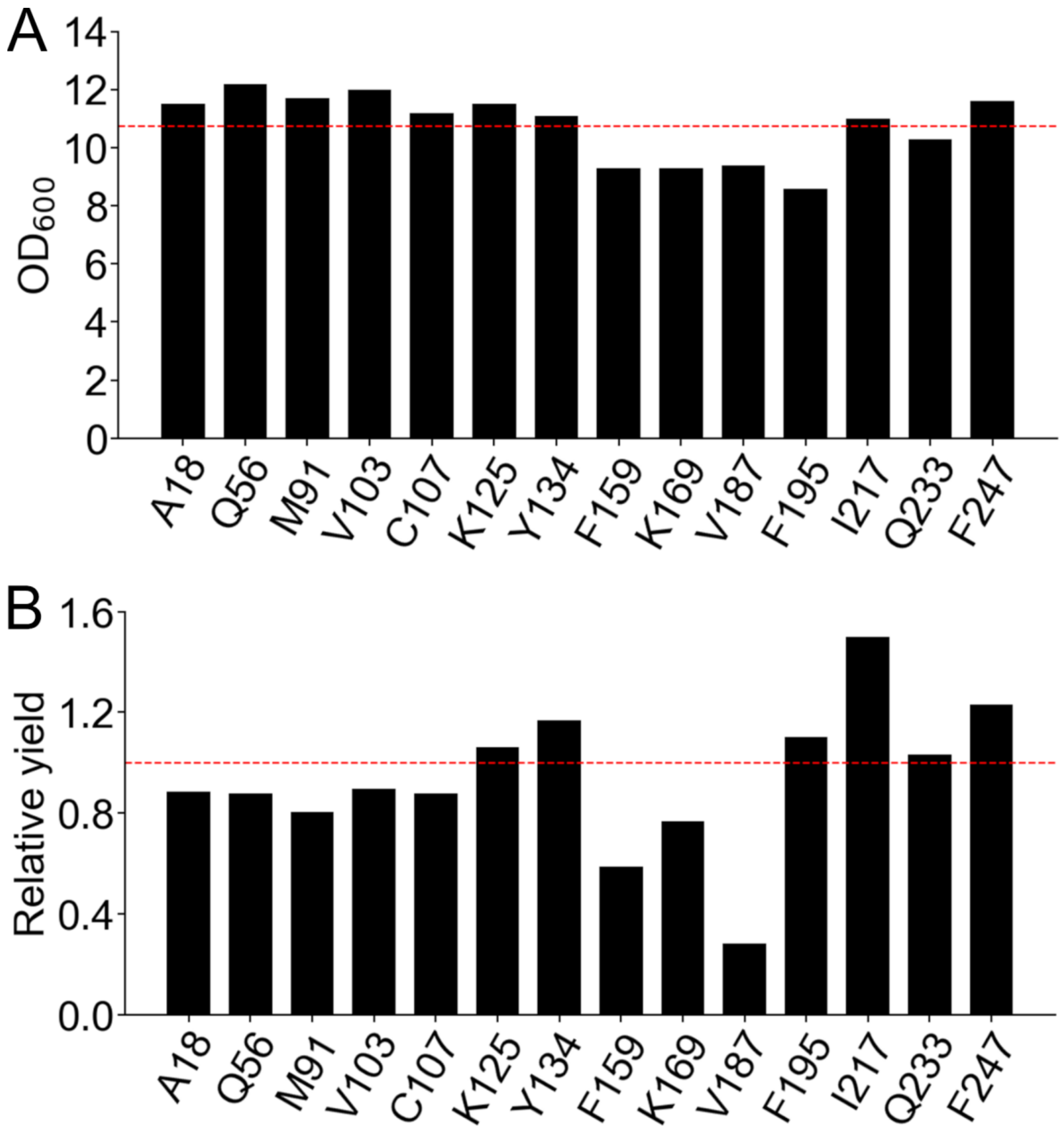

Figure S2. The protein yield of hH_v1-Acd depended on the position of Acd incorporation. (A) Optical density at 600 nm (OD_{600}) of the final cultures expressing the hH_v1 with Acd replacing the indicated amino acid. (B) Relative protein yield calculated by multiplying the OD_{600} and Western blot band intensity of cellular extracts from the final cultures expressing the hH_v1 with Acd replacing the indicated amino acid. Values of normalized by the No TAG condition, which are cells expressing the hH_v1 without any amber stop codon in the presence of Acd and the aminoacyl-tRNA synthetase/tRNA pair (red broken line).

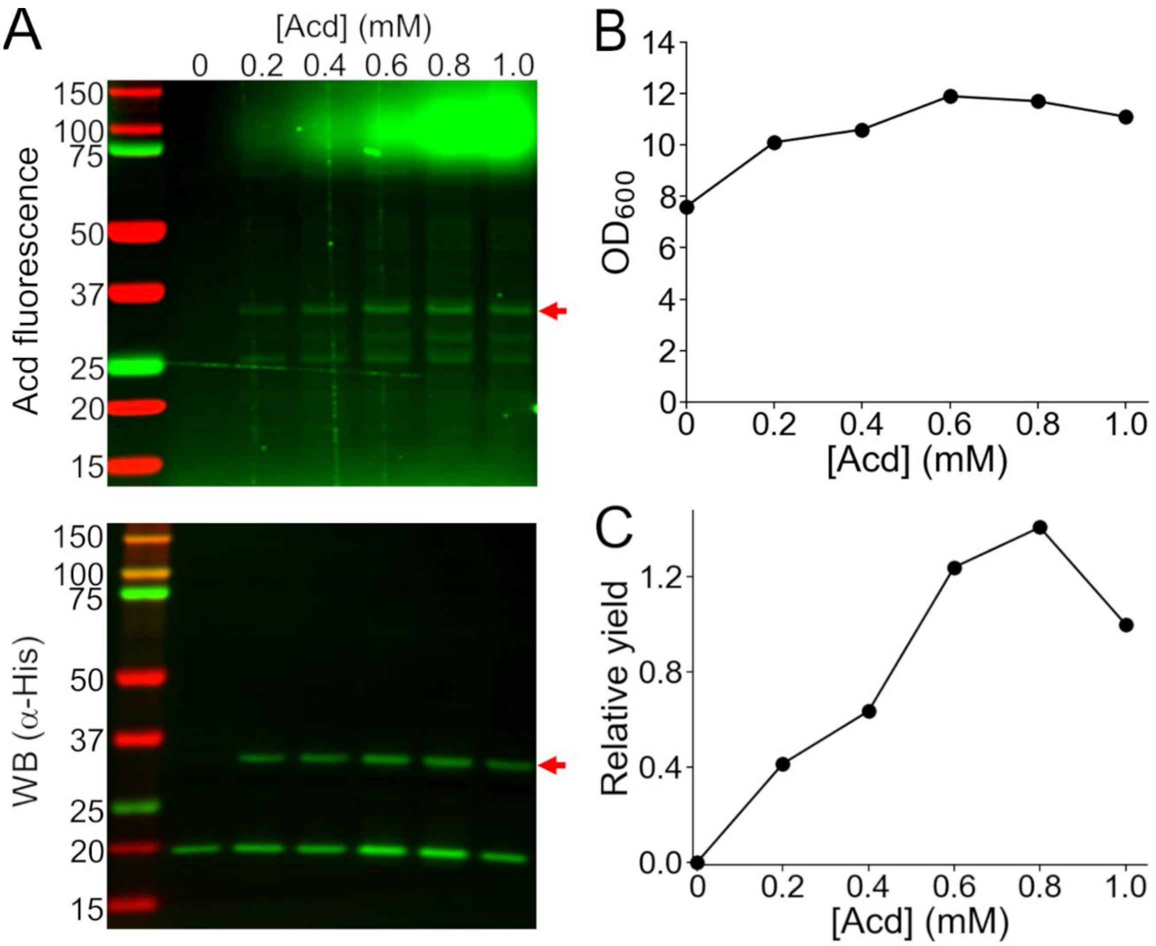

Figure S3. The truncation of hH$_v$1-K125Acd was not decreased at higher concentrations of Acd in the culture medium. (A) Cellular extracts of cells co-transformed with the MjA9-AcdRS/tRNA pair plasmid and hH$_v$1-K125TAG and grown with the indicated concentration of Acd in the culture medium were separated by SDS-PAGE to visualize the hH$_v$1 band (red arrow) by Acd fluorescence (top) and Western blot against the N-terminus His-tag (bottom). (B) Optical density at 600 nm (OD_{600}) of the cultures at the end of the expression. (C) Relative yield obtained by multiplying the Western blot band intensity by the OD600, normalized by the value obtained at 1 mM Acd.

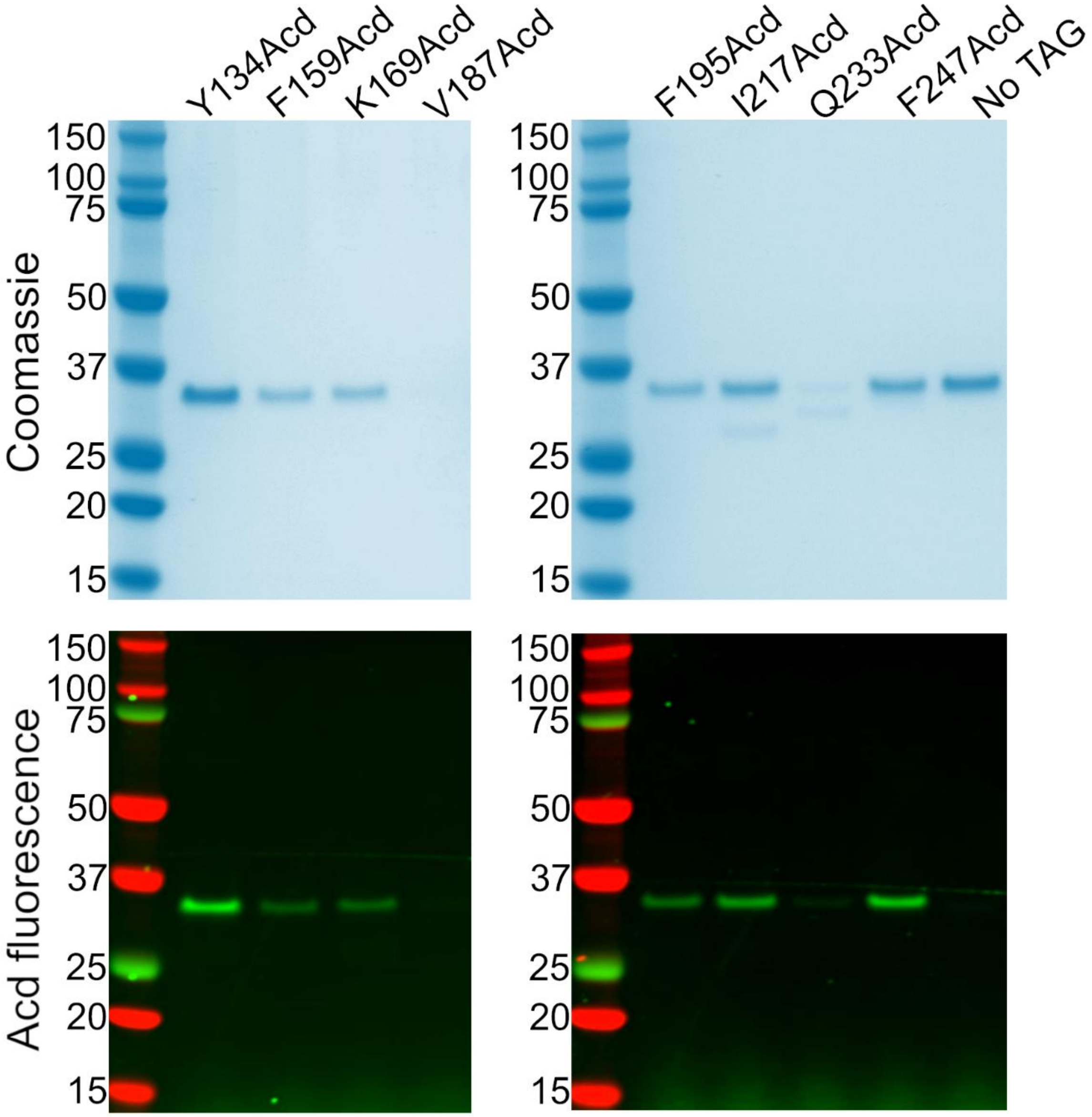

Figure S4. SDS-PAGE of the hH$_v$1 protein samples after purification. Coomassie-stained (top) and Acd fluorescence (bottom) gels of the samples from the elution of the nickel resin column, separated by SDS-PAGE. Note that V187Acd and Q233Acd did not contain appreciable amounts of protein. The No TAG condition consisted of samples purified from cells expressing the hH$_v$1 without any amber stop codon in the presence of Acd and the aminoacyl-tRNA synthetase/tRNA pair.

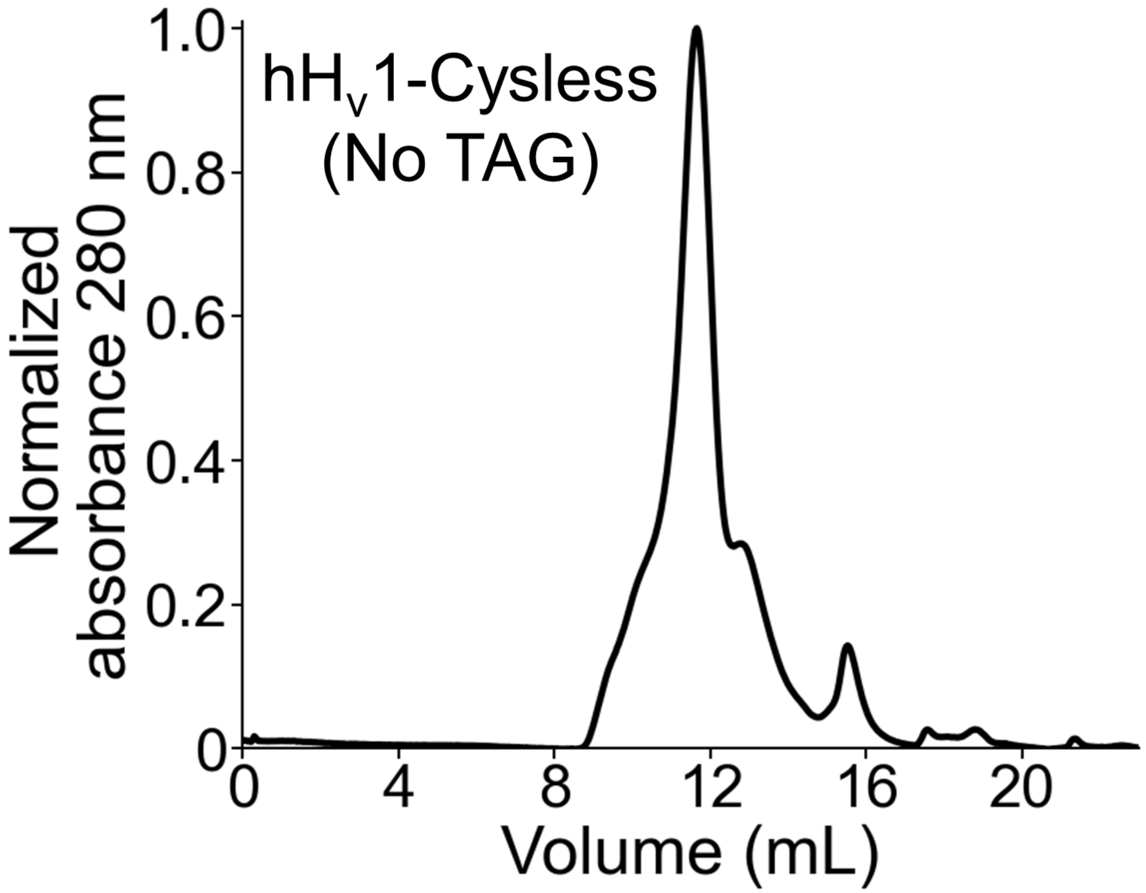

Figure S5. Size exclusion chromatogram of the No TAG sample. The No TAG sample was purified from cells expressing the hH_v1 without any amber stop codon in the presence of Acd and the aminoacyl-tRNA synthetase/tRNA pair. The sample eluted from the immobilized affinity chromatography column was concentrated and injected into the size exclusion chromatography column.

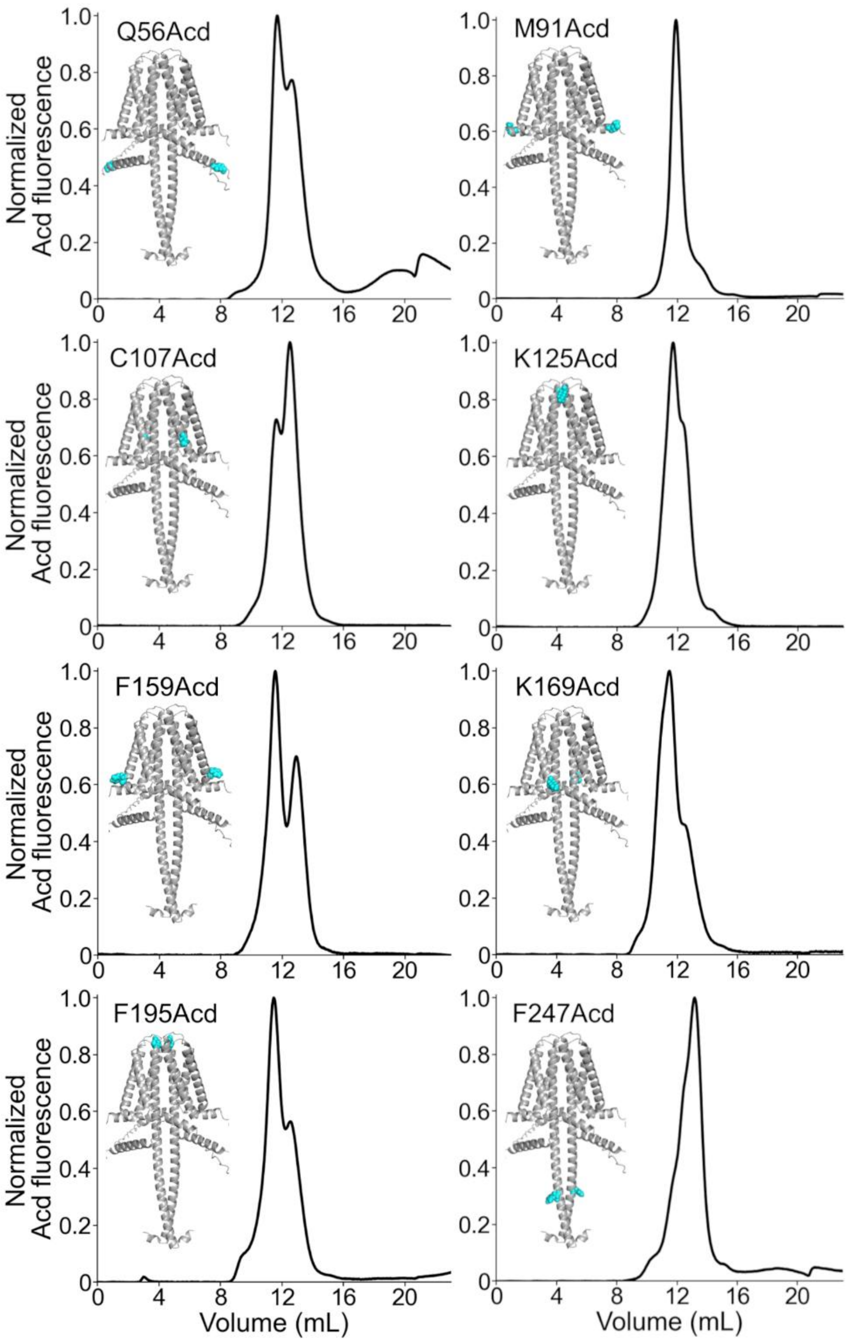

Figure S6. Fluorescence-detection size exclusion chromatography of hH$_v$1-Acd proteins. Chromatograms of the purified hH$_v$1 proteins with Acd incorporated at the indicated amino acid position. The AlphaFold dimer model with the amino acid replaced by Acd as cyan spheres is included.

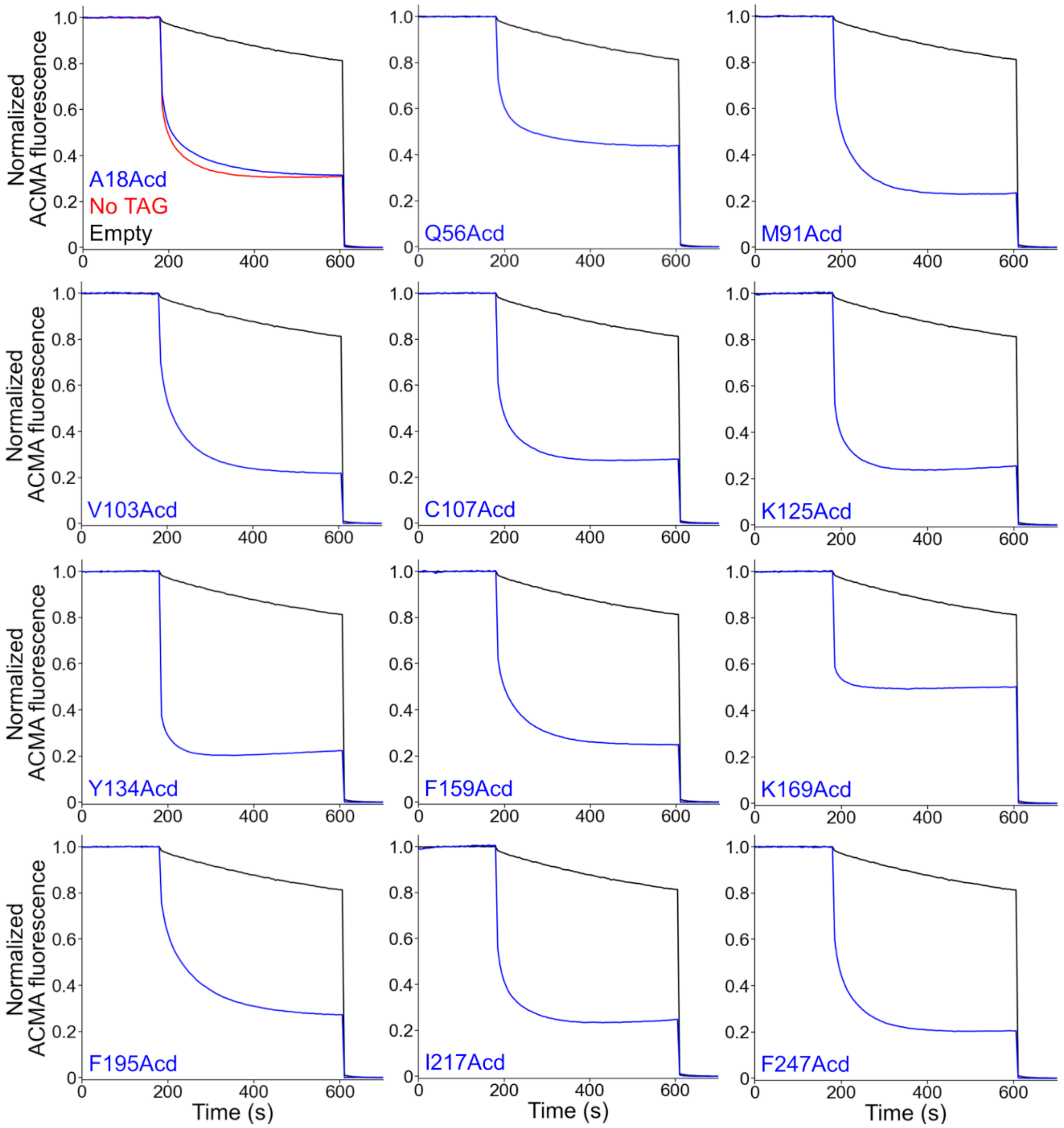

Figure S7. The purified hH_v1-Acd proteins were functional proton channels. Liposome proton flux assays of asolectin proteoliposomes containing the indicated hH_v1-Acd protein. The proteoliposome samples produced ACMA fluorescence quenching after the addition of valinomycin (180 s) in the presence of a potassium gradient. The protonophore CCCP was added at the end of the experiment (600 s). The No TAG sample (red trace) contains proteoliposomes containing hH_v1 without any amber stop codon expressed in the presence of Acd and the aminoacyl-tRNA synthetase/tRNA pair. Empty asolectin liposomes (black traces) showed slow ACMA fluorescence quenching.

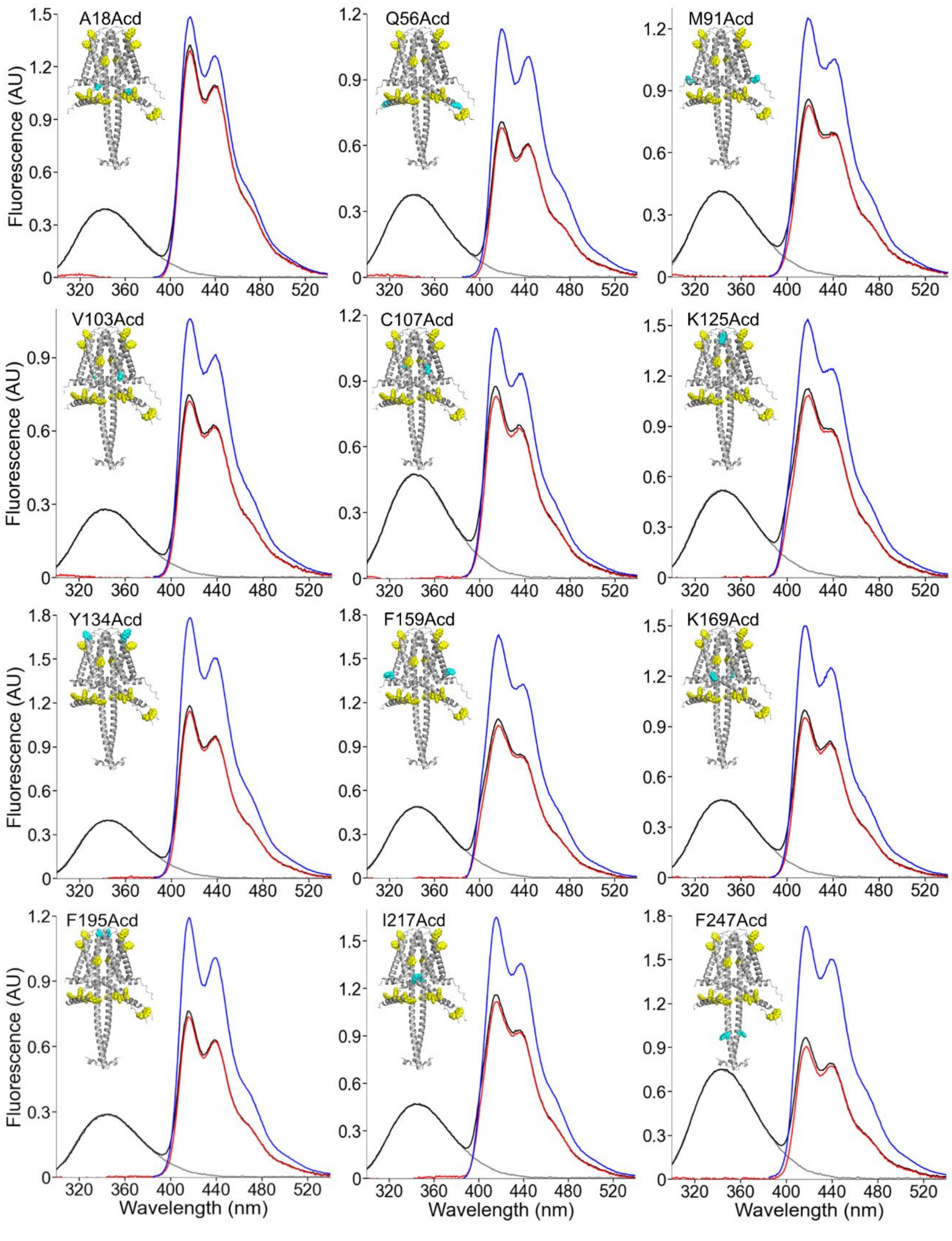

Fig. S8. Fluorescence emission spectra of hH$_V$1-Acd. Spectra of the indicated hH$_V$1-Acd protein sample obtained when exciting at 280 nm (black) and 370 nm (blue). The isolated spectrum of Acd (red) was obtained by subtracting the black trace from the normalized spectrum of the No TAG sample excited at 280 nm (grey).

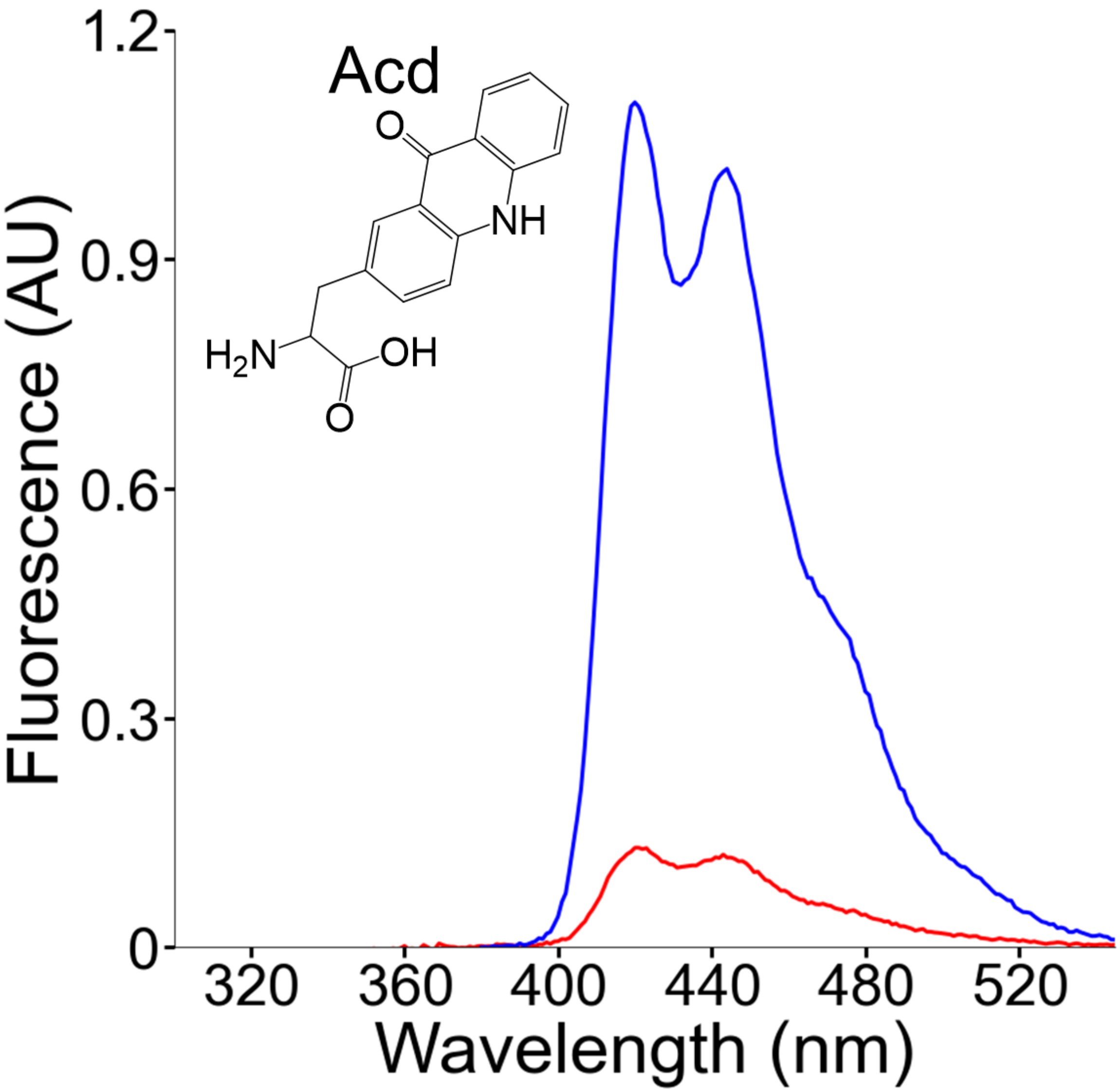

Figure S9. Fluorescence emission spectra of the Acd amino acid in solution. Spectra of Acd in Buffer-H2 using an excitation wavelength of 370 nm (blue) or 280 nm (red).

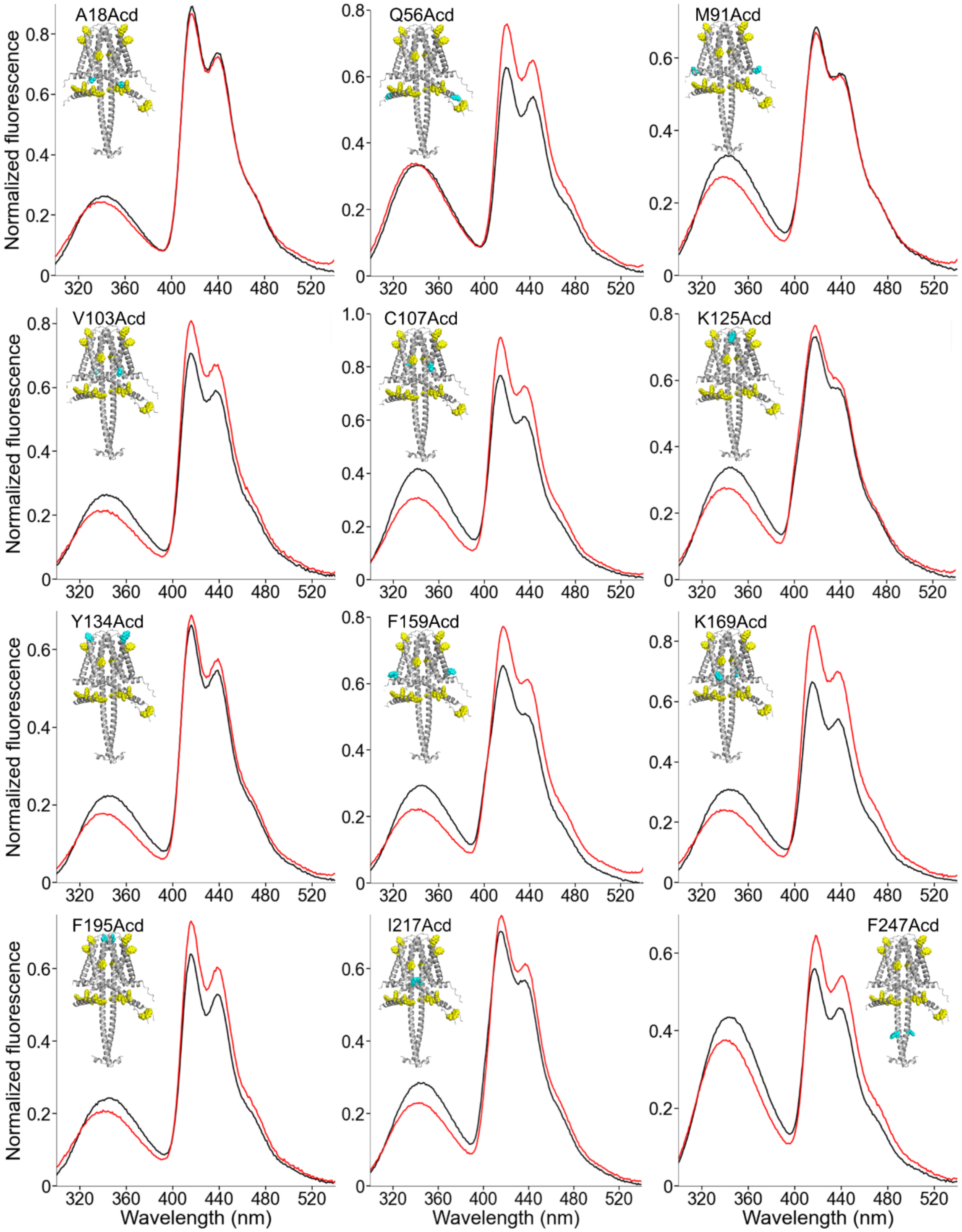

Fig. S10. Changes in the fluorescence emission spectra of hH$_v$1-Acd in the presence of zinc. Spectra of the indicated hH$_v$1-Acd protein sample obtained when exciting at 280 nm in the Apo state (black) and in the presence of 1 mM Zn^{2+} (red). Spectra were normalized by the maximum intensity of the Acd emission spectrum of the same sample excited at 370 nm.

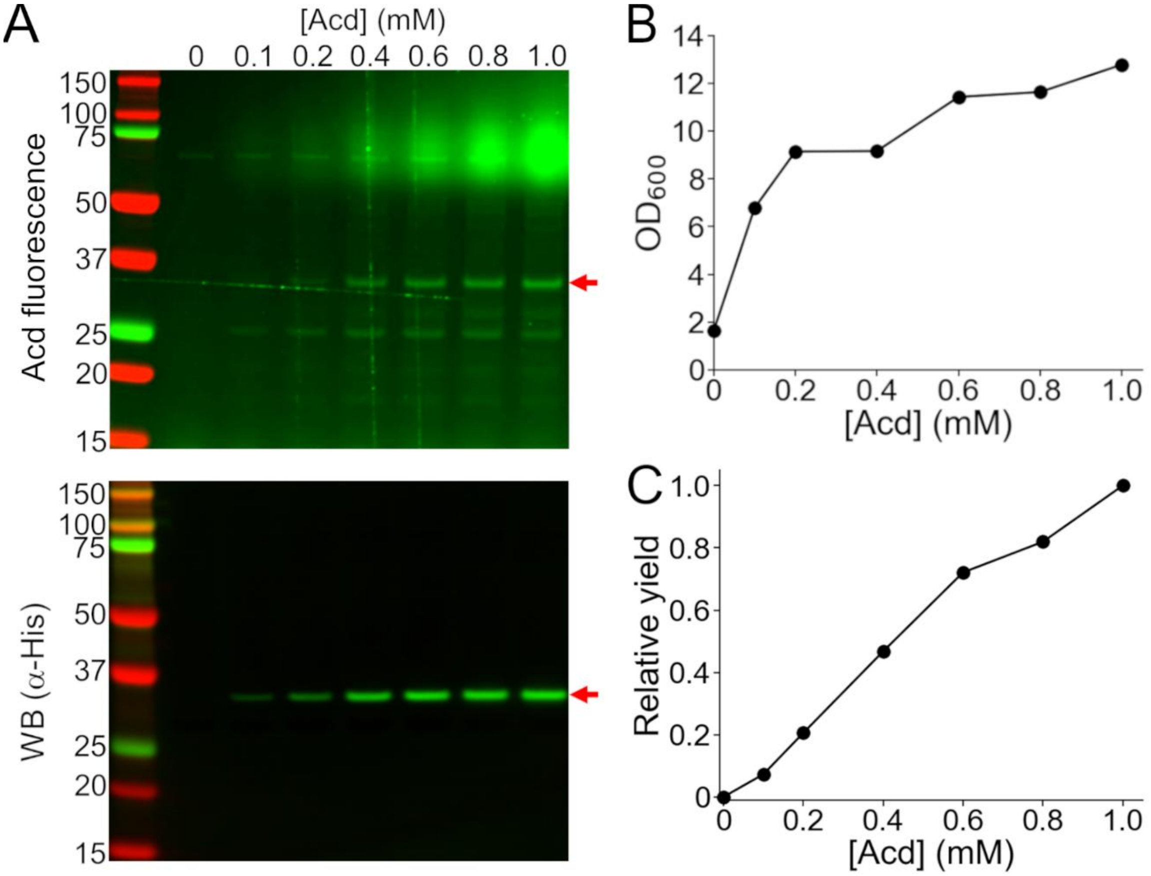

Figure S11. The protein yield of hH$_v$1-A18Acd was proportional to the concentration of Acd in the culture medium. (A) Cellular extracts of cells co-transformed with the MjA9-AcdRS/tRNA pair plasmid and hH$_v$1-A18TAG and grown with the indicated concentration of Acd in the culture medium were separated by SDS-PAGE to visualize the hH$_v$1 band (red arrow) by Acd fluorescence (top) and Western blot against the N-terminus His-tag (bottom). (B) Optical density at 600 nm (OD_{600}) of the cultures at the end of the expression. (C) Relative yield obtained by multiplying the Western blot band intensity by the OD_{600}, normalized by the value obtained at 1 mM Acd.

Supplementary Tables

Table S1. Size-exclusion chromatography elution volumes of the hH_v1 proteins. Elution volumes were measured as the location of the maximum value of absorbance at 280 nm in the chromatograms.

hH_v1 sample	Elution volume (mL)
No TAG	11.5
A18Acd	11.6
Q56Acd	11.7
M91Acd	11.9
V103Acd	11.5
C107Acd	12.5
K125Acd	11.7
Y134Acd	11.5
F159Acd	11.5
K169Acd	11.5
F195 Acd	11.5
I217Acd	12.4
F247Acd	13.1

Table S2. Emission fluorescence spectrum maximum of Acd in different solvents. The wavelength corresponds to the location of the maximum intensity of the emission fluorescence spectrum of the free amino acid dissolved in the corresponding solvent. EtAc: ethyl acetate, OctOH: 1-octanol, ButOH: 1-butanol, EtOH: ethanol.

**Solvent**	**Emission Maximum (nm)**
EtAc	402
OctOH	414
ButOH	415
EtOH	414
Water	421
Buffer-H2	420

Table S3. The emission fluorescence spectrum maximum of the hH_v1-Acd proteins. The wavelength corresponds to the location of the maximum intensity of the emission fluorescence spectrum of the samples.

hH_v1 sample	Emission Maximum (nm)
A18Acd	418
Q56Acd	420
M91Acd	419
V103Acd	417
C107Acd	415
K125Acd	418
Y134Acd	416
F159Acd	418
K169Acd	415
F195 Acd	416
I217Acd	415
F247Acd	418

Supplementary References

1. J. R. Lakowicz, Ed., *Principles of Fluorescence Spectroscopy* (Springer US, 2006).

2. M. H. Tessmer, S. Stoll, chiLife: An open-source Python package for in silico spin labeling and integrative protein modeling. *PLoS Comput Biol* **19**, e1010834 (2023).
